# The Morphological Transformation of the Thorax during the Eclosion of *Drosophila melanogaster* (Diptera: Drosophilidae)

**DOI:** 10.3390/insects14110893

**Published:** 2023-11-18

**Authors:** Si-Pei Liu, Hao-Dong Yin, Wen-Jie Li, Zhuang-Hui Qin, Yi Yang, Zheng-Zhong Huang, Le Zong, Xiao-Kun Liu, Zhong Du, Wei-Li Fan, Ya-Qiong Zhang, Dan Zhang, Yong E. Zhang, Xing-Yue Liu, Ding Yang, Si-Qin Ge

**Affiliations:** 1 Key Laboratory of Zoological Systematics and Evolution, Institute of Zoology, Chinese Academy of Sciences, Beijing 100101, China; spliu@ioz.ac.cn (S.-P.L.); haodong.yin@okstate.edu (H.-D.Y.); liwenjie@ioz.ac.cn (W.-J.L.); qinzh2023@163.com (Z.-H.Q.); yangyi@ioz.ac.cn (Y.Y.); huangzz@ioz.ac.cn (Z.-Z.H.); zongle@ioz.ac.cn (L.Z.); liuxiaokun22@ioz.ac.cn (X.-K.L.); dzzz159753@outlook.com (Z.D.); 15110637937@163.com (W.-L.F.); zhangyq@ioz.ac.cn (Y.-Q.Z.); classmate.s@163.com (D.Z.); zhangyong@ioz.ac.cn (Y.E.Z.); 2 University of Chinese Academy of Sciences, Beijing 100086, China; 3 Shanghai Frontiers Science Center of Genome Editing and Cell Therapy, Shanghai Key Laboratory of Regulatory Biology, Institute of Biomedical Sciences and School of Life Sciences, East China Normal University, Shanghai 200062, China; 4 Department of Entomology, College of Plant Protection, China Agricultural University, Beijing 100193, China; xingyue_liu@yahoo.com (X.-Y.L.); dyangcau@126.com (D.Y.)

**Keywords:** *Drosophila melanogaster*, metamorphosis, eclosion, thorax, anatomical morphology

## Abstract

**Simple Summary:**

The developmental process, divided into four different stages (egg, larva, pupa and adult), is the main reason for their remarkable diversification and expansion of the insect group Holometabola. Advanced morphological techniques have been used to demonstrate the 3D thoracic anatomical structures of the holometalous model organism fruit fly, *Drosophila melanogaster*, before and after emergence, in order to uncover the transformation process of the muscles, nerves, and gut during development. Skeletal changes affect the original positions of the muscles. The muscles vary in size, not only becoming longer and broader, but also shorter and narrower. Different muscle shapes may appear during development. The number of bundles may also vary. The soft tissues in the body may fix the free ends of the growing muscles, and a strong adult skeleton likely causes the absence of some muscles and tendons. The flight muscles appear very early, probably to achieve full functionality of these very large adult-specific muscles in time. There are some differences during the same developmental period between the two sexes. Most muscles of the larvae and adults with similar attachment positions change their functions from supporting crawling to supporting flying and walking under the control of a more complex ventral nerve cord. The midguts of the larva and the adult are nearly the same.

**Abstract:**

The model organism *Drosophila melanogaster*, as a species of Holometabola, undergoes a series of transformations during metamorphosis. To deeply understand its development, it is crucial to study its anatomy during the key developmental stages. We describe the anatomical systems of the thorax, including the endoskeleton, musculature, nervous ganglion, and digestive system, from the late pupal stage to the adult stage, based on micro-CT and 3D visualizations. The development of the endoskeleton causes original and insertional changes in muscles. Several muscles change their shape during development in a non-uniform manner with respect to both absolute and relative size; some become longer and broader, while others shorten and become narrower. Muscular shape may vary during development. The number of muscular bundles also increases or decreases. Growing muscles are probably anchored by the tissues in the stroma. Some muscles and tendons are absent in the adult stage, possibly due to the hardened sclerites. Nearly all flight muscles are present by the third day of the pupal stage, which may be due to the presence of more myofibers with enough mitochondria to support flight power. There are sexual differences in the same developmental period. In contrast to the endodermal digestive system, the functions of most thoracic muscles change in the development from the larva to the adult in order to support more complex locomotion under the control of a more structured ventral nerve cord based on the serial homology proposed herein.

## 1. Introduction

Holometabola, which comprises nearly 85% of insect diversity, includes more species than there are among plants or all other combined animal phyla [1]. Insects of Holometabola undergo an extreme form of metamorphosis that comprises a series of discrete stages (egg, larva, pupa and adult), during which organs and tissues are extensively remodeled and even completely rebuilt [2,3]. This transformation, regulated mainly by juvenile hormone and ecdysone [4,5], is important from an ecological viewpoint, as larvae and adults are able to occupy different ecological niches [6]. Therefore, metamorphosis is considered one of the reasons for the tremendous success of Holometabola in terms of its diversification and expansion [7,8].

For over one hundred years, *Drosophila melanogaster* (Diptera: Drosophilidae), commonly known as the fruit fly, has been used for both medical and scientific research as a model organism [9]. Roughly, it undergoes a 24 h embryonic development, a 4-day larval stage with three instars, and a 5-day pupal stage under optimal conditions [10]. There is considerable interest in the changes experienced by the locomotion-related organs in the thorax during the pupal stage, and particularly in the formation of wings of *Drosophila*. Relevant studies refer to the development of veins [11], genetic control of force patterns in developing wings [12], or fold formation at the wing boundary [13]. The skeleton and muscles in the thorax provide power for wing flapping. Research on the morphological changes in the thorax of *D. melanogaster* during metamorphosis using light microscopes has a long history.

Robertson [14] recorded the development of imaginal discs, the related hypodermis, and structures including the legs, wings, prothoracic spiracles, bristles, and hairs. He found that larva muscles were destroyed by histolysis and consumed by phagocytes during prepupal and early pupal instars, and differentiation into the imaginal muscles could be seen in 21 h pupa. A sequence of 51 visible changes during metamorphosis were observed by Bainbridge and Bownes [15]. They mentioned changes in thoracic structure, including extension of the legs and wings, the movement of Malpighian tubules from the thorax into the abdomen, the formation of bristles, and the darkening of wing color. Bodenstein [16] and Hartenstein [17] reviewed the process of postembryonic development from all aspects. These studies either focus more on physiological processes, or do not provide sufficient details about morphological changes involved in the growth from pupa to adult; there is an absence of descriptions of the changes in each muscle on each day. Zalokar [18], Ferris [19], and Miller [20] described the skeleton and the musculature of the adult through hand-drawings. Fabian et al. [21] studied the comparative thoracic anatomy of wild-type and wingless mutant of *D. melanogaster* based on advanced morphological techniques. They also listed homologous muscles based on their own studies and those of Zalokar [18] and Miller [20].

In this study, more detailed anatomical transformations of the thorax during the process of eclosion from the late pupal stage to after the emergence of the wild-type iso-1 of *D. melanogaster* were recorded using micro-CT and computer-based 3D visualization, and are discussed herein. We also infer the serial homology of the larva to the adult according to a comparison of the anatomical structure of the third instar larva [22] with our results. Currently, the online database of the Drosophilidae family FlyBase (www.flybase.org, accessed on 26 September 2023) [23] has information from a variety of sources, and facilitates the discovery of significant relationships among them. We hope the morphological findings of our study will provide some novel insights.

## 2. Materials and Methods

### 2.1. Raising Specimens

Wild-type iso-1 *Drosophila melanogaster* adults were provided by the Group of Computational Evolutionary Genomics, IOZCAS, and raised in a laboratory culture maintained at a constant temperature of 25 ± 1 °C and 60% relative humidity under a 12 h–12 h light–dark cycle. Eggs were collected 2 h after adult mating and then kept in a glass container. The gonad located in the penultimate segment of the abdomen of the third instar larva was observed with a microscope to distinguish between males and females. After that, the separated male and female larvae were transferred to two incubators, respectively. The larvae and adults were fed growth medium with preservative (Table 1).

### 2.2. Specimen Collection

After the prepupal stage, ten pupal specimens of each male and female were collected every day until emergence, with each set labeled by the day of collection (D1 to D5). Then, ten adult specimens of each male and female were collected each day from hatching to the third day (D1 to D3). All the specimens were preserved in 75% ethanol.

### 2.3. Microcomputer Tomography and Computer-Based 3D Reconstruction

**Figure 1:**
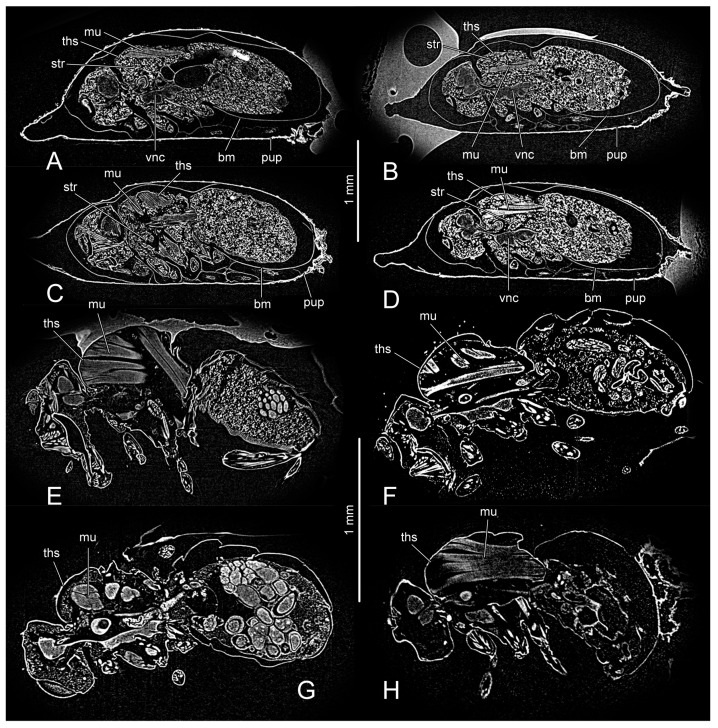
*Drosophila melanogaster*, micro-CT scans. Female: (**A**): PD3; (**C**): PD4; (**E**): AD1; (**G**): AD3. Male: (**B**): PD3; (**D**): PD4; (**F**): AD1; (**H**): AD3. The scale bar above refers to (**A**–**D**); the scale bar below refers to (**E**–**H**). Abbreviations: bm—basal membrane; mu—muscles; pup—puparium; str—stroma; ths—thoracic skeleton; vnc–ventral nerve cord.

The third day and the fourth day pupae and the first day and the third day adults were dehydrated in an ascending ethanol series (75%–85%–90%–95%–100%) and dried at the critical point (Leica EM CPD300, IOZCAS, Beijing, China). They were scanned using a micro-CT (Zeiss microXCT-400, IOZCAS, Beijing, China). The thoracic part of each specimen was reconstructed three-dimensionally based on a micro-CT image stack (Figure 1) using Amira 6.0 (Thermo Fisher Scientific, Waltham, MA, USA). The parameters of the 3D reconstructions used here are listed in Table 2. Segmented structures were exported as stacks of tiff files into VG Studio Max 3.0 (Volume Graphics, Heidelberg, Germany) for volume rendering. The final images were edited and hand-drawn based on 3D reconstructions using Adobe Illustrator 2017 (Adobe Inc., Mountain View, CA, USA).

### 2.4. Terminology and Abbreviations

The terminology for the endoskeleton follows the work of Fabian et al. [21]; for muscles, the work of Friedrich and Beutel [24]; for the nervous system, the work of Court et al. [25]; and for the digestive system, that of Miller [20]. PD3 and PD4 are used to represent, respectively, the third and fourth days of the pupal stage, and AD1 and AD3 the first and third days of the adult stage.

## 3. Results

**Figure 2:**
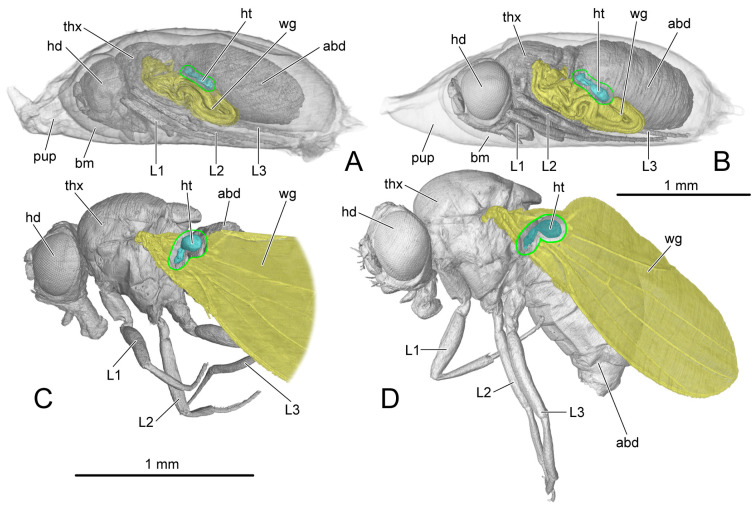
Whole bodies of *Drosophila melanogaster* from the late pupal stage to after emergence, represented using a 3D visualization. The exoskeletons are colored light grey. The wings are colored light yellow. The halteres are colored blue. Parts of the wings are removed to show the halteres surrounded by green lines. (**A**): PD3; (**B**): PD4; (**C**): AD1; (**D**): AD3. The scale bar above refers to (**A**,**B**); the scale bar below refers to (**C**,**D**). Abbreviations: abd—abdomen; bm—basal membrane; hd—head; ht—halter; L1/2/3—foreleg/midleg/hindleg; pup—puparium; thx—thorax; wg—wing.

The thoracic skeleto-muscular system, nervous system, and digestive system of PD3, PD4, AD1, and AD3 are used to represent the eclosion process of *D. melanogaster* (Figure 2). In the pupal stage, the insect body is covered by the basal membrane (bm: Figure 1A–D and Figure 2A,B) and puparium (pup: Figure 1A–D and Figure 2A,B). A high concentration of stroma (str: Figure 1A–D), which is very bright upon micro-CT, fills the body cavity.

### 3.1. Skeletons

**Figure 3:**
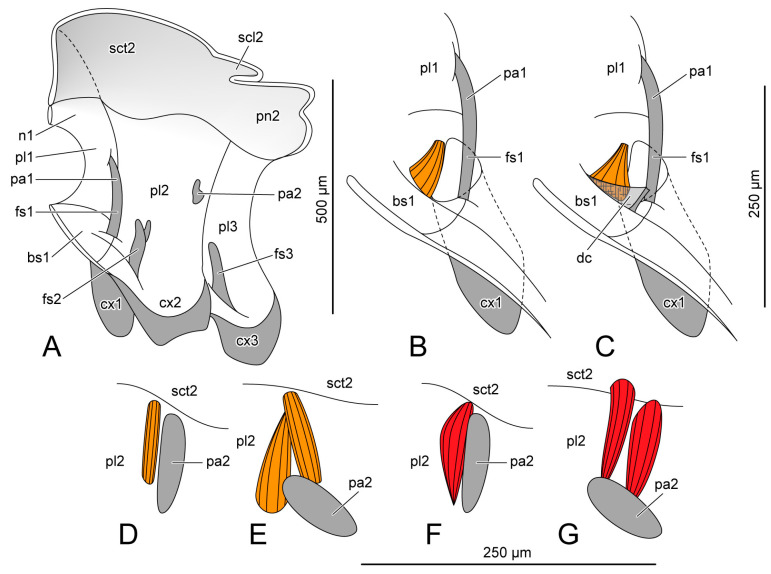
Hand-drawings of endoskeletal and muscular structures. (**A**): female endoskeleton on PD3, sagittal section; (**B**): the central area of the probasisternum, of Iscm1 origin; (**C**): the discrimen of the probasisternum, of Iscm1 origin; (**D**): male IItpm8 on PD3; (**E**): male IItpm8 on PD4; (**F**): male IItpm9 on PD3; (**G**): male IItpm9 on PD4. The scale bar on the upper left refers to (**A**); the scale bar on the upper right refers to (**B**,**C**); the scale bar below refers to (**D**–**G**). Abbreviations: bs1—probarsisternum; cx1/2/3—pro-/meso-/metacoxa; dc—discrimen; fs1/2/3—pro-/meso-/metafurca; n1—pronotum; pa1/2—pro-/mesopleural apophysis; pl1/2/3—pro-/meso-/metapleuron; pn2—mesopostnotum; scl2—mesoscutellum; sct2—mesoscutum.

On PD3 and PD4, the wings (wg: Figure 2A,B) are strongly folded to fit the narrow space in the puparium, and the halteres are close to the body (ht: Figure 2A,B). On AD1 and AD3, the wings (wg: Figure 2C,D) spread, and the halteres raised (ht: Figure 2C,D). The male and female endoskeletons are similar. Here, we present the female endoskeletons, for which the 3D visualization is better quality than that for the male. The thoracic skeleton in the pupal stage (ths: Figure 1A–D) is delicate and difficult to distinguish from the stroma in the micro-CT results; therefore, we represent the endoskeleton on PD3 using hand-drawings (Figure 3A). The thoracic length is measured from the anterior margin of the mesoscutum to the posterior margin of the mesopostnotum (Table 3).

#### 3.1.1. Prothoracic Skeletons

**Figure 4:**
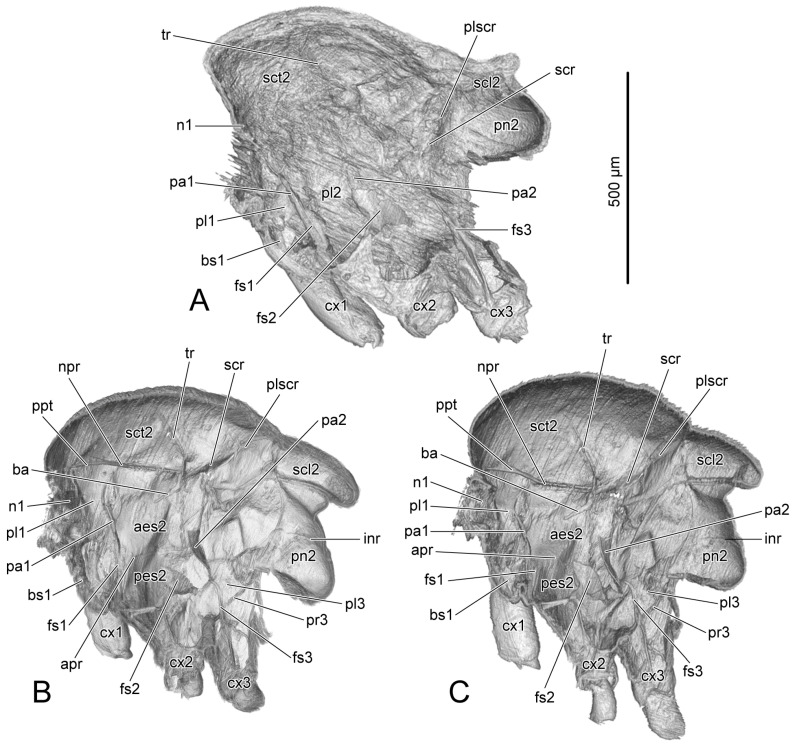
The female endoskeletons of *Drosophila melanogaster*, 3D visualization. (**A**): PD4; (**B**): AD1; (**C**): AD3. Abbreviations: aes2—mesanepisternum; apr—anapleural ridge; ba—mesobasalare; bs1—probasisternum; cx1/2/3—pro-/meso-/metacoxa; fs1/2/3—pro-/meso-/metafurca; n1—pronotum; npr—notopleural ridge; pa1/2—pro-/mesopleural apophysis; pes2—mesopreepisternum; pl1/2/3—pro-/meso-/metapleuron; plscr—posterolateral scutal ridge; pn2—mesopostnotum; ppt—postpronotal trabecle; pr3—metapleural ridge; scl2—mesoscutellum; scr—scutal ridge; sct2—mesoscutum; tr—transverse ridge.

PD3 (Figure 3A): The prothorax has formed the dorsal pronotum (n1: Figure 3A), the ventral probasisternum (bs1: Figure 3A), and the lateral propleuron (pl1: Figure 4A) as the attachment position for the prothoracic muscles. The propleural apophysis (pa1: Figure 3A) extends ventro-proximally from the posterior margin of the propleuron to connect with the profurca (fs1: Figure 3A), located in in postero-lateral margin of the probasisternum. The probasisternum has no central discrimen. The procoxal muscle, Iscm1, directly connects with the central area of the probasisternum (Figure 3B).

PD4 (Figure 4A): In the female, a discrimen (dc: Figure 3C) is formed on the central line of the probasisternum, whereas the male does not have this structure.

AD1 (Figure 4B): Both the male and female have a discrimen of probasisternum, connected to Iscm1.

AD3 (Figure 4C): The prothoracic structure on AD3 is the same as that on AD1.

#### 3.1.2. Mesothoracic Skeletons

PD3 (Figure 3A): The dorsal area of the mesothorax is divided into the anterior mesoscutum (sct2: Figure 3A), the postero-central mesoscutellum (scl2: Figure 3A), and the posterior mesopostnotum (pn2: Figure 3A). The anterior area of the mesoscutum slightly bends backwards. The small and flat mesoscutellum has almost no interior space; therefore, the mesoscutellar muscle, IIdlm3, is absent in both the male and female. The posterior area of the mesopostnotum concaves forwards. The female mesopleural apophysis (pa2: Figure 3A) is a small process that occurs in the posterior area of the mesopleuron (pl2: Figure 3A). The male mesopleural apophysis is slightly larger. Ventrally, on the central margin anterior to the mesocoxa (cx2: Figure 3A), the mesofurca (fs2: Figure 3A) extends upwards with a forked dorsal part. Most endoskeletal ridges do not develop well, or cannot be recognizable from the micro-CT results, due to the soft and delicate skeleton being difficult to distinguish from the stroma.

PD4 (Figure 4A): Both the mesoscutellum (sct2: Figure 4A) and mesopostnotum (pn2: Figure 4A) enlarge and enclose more inner space. The muscle IIdlm3 is present in both the male and female. The female mesopleural apophysis (pa2: Figure 4A) enlarges. The male mesopleural apophysis rotates forwards (Figure 3D–G). The dorsal area of the mesofurcal arm (fs2: Figure 4A) enlarges to form a pair of pallets connected to the muscle IIspm2. There are the lateral scutal ridge (scr: Figure 4A) and the postero-lateral posterolateral scutal ridge (plscr: Figure 4A), which delimit the mesoscutellum from the meoscutum. The transverse ridge (tr: Figure 4A) appears across the mesoscutum.

AD1 (Figure 4B): The anterior area of the mesoscutum (sct2: Figure 4B) projects forwards. Both the mesoscutellum (scl2: Figure 4B) and the mesopostnotum (pn2: Figure 4B) enlarge. The latter extends postero-ventrally. There is a slender mesobasalare (ba: Figure 4B) extending antero-ventrally from the postero-dorsal edge of the mesanepisternum to connect to the muscle IItpm3. The mesopleural apophysis (pa2: Figure 4B) enlarges to form a long triangular shape. The new endoskeletal structures include the postpronotal trabecle (ppt: Figure 4B), separating the mesoscutum and propleuron; the notopleural ridge (npr: Figure 4B), separating the mesoscutum and mesanepisternum; the anapleural ridge (apr: Figure 4B), separating the anterior area of the mesopleuron into the dorsal mesanepisternum (aes2: Figure 4B) and the ventral mesopreepisternum (pes2: Figure 4B); and the internotal ridge (inr: Figure 4B) across the mesopostnotum.

AD3 (Figure 4C): The mesothoracic structure on AD3 is same as that on AD1.

#### 3.1.3. Metathoracic Skeletons

PD3 (Figure 3A): Below the mesopostnum, there is the narrow metathorax. The metafurca (fs3) extends antero-dorsally from the anterior margin of the metacoxal (cx3: Figure 3A).

PD4 (Figure 4A): A forked structure is formed on the dorsal area of the metafurca (fs3: Figure 4A).

AD1 (Figure 4B): The metapleural ridge (pr3: Figure 4B) extends from the postero-dorsal margin of the metapleuron to the antero-lateral margin of the metacoxal rim.

AD3 (Figure 4C): The metathoracic structure on AD3 is same as that on AD1.

### 3.2. Muscles

**Figure 5:**
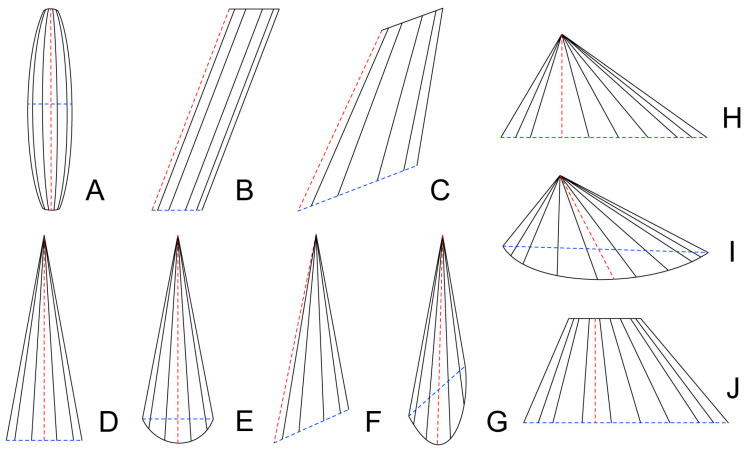
Measurement rules for muscles with different shapes. Lengths are denoted with red dotted lines. Widths are denoted with blue dotted lines. (**A**): broad medially and narrowing towards both ends; (**B**): parallelogram; (**C**): approximate parallelogram; (**D**): isosceles long triangle; (**E**): approximate long isosceles triangle; (**F**): long triangle; (**G**): approximate long triangle; (**H**): flat triangle; (**I**): approximate flat triangle; (**J**): trapezoid.

**Figure 6:**
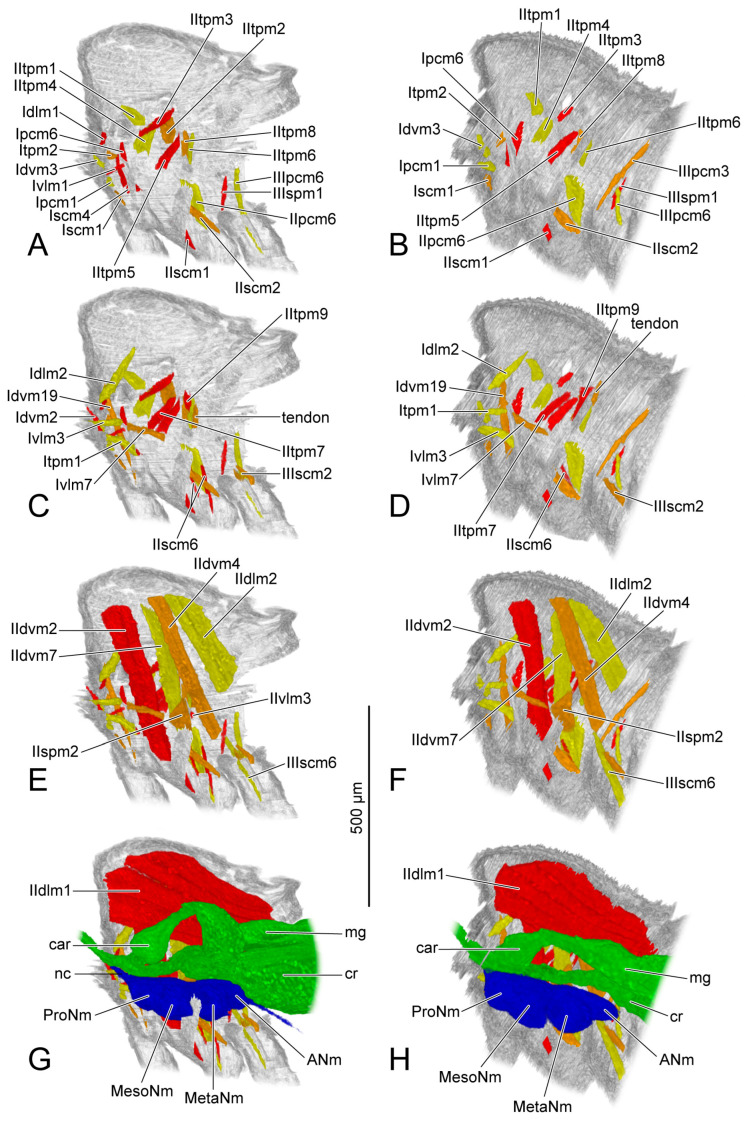
Musculature of *Drosophila melanogaster* in the PD3 sagittal section. Female: (**A**,**C**,**E**,**G**); Male: (**B**,**D**,**F**,**H**). Abbreviations: ANm–abdominal neuromere; car—cardia; cr—crop; MesoNm–mesothoracic neuromere; MetaNm–metathoracic neuromere; mg—midgut; nc—neck connective.

**Figure 7:**
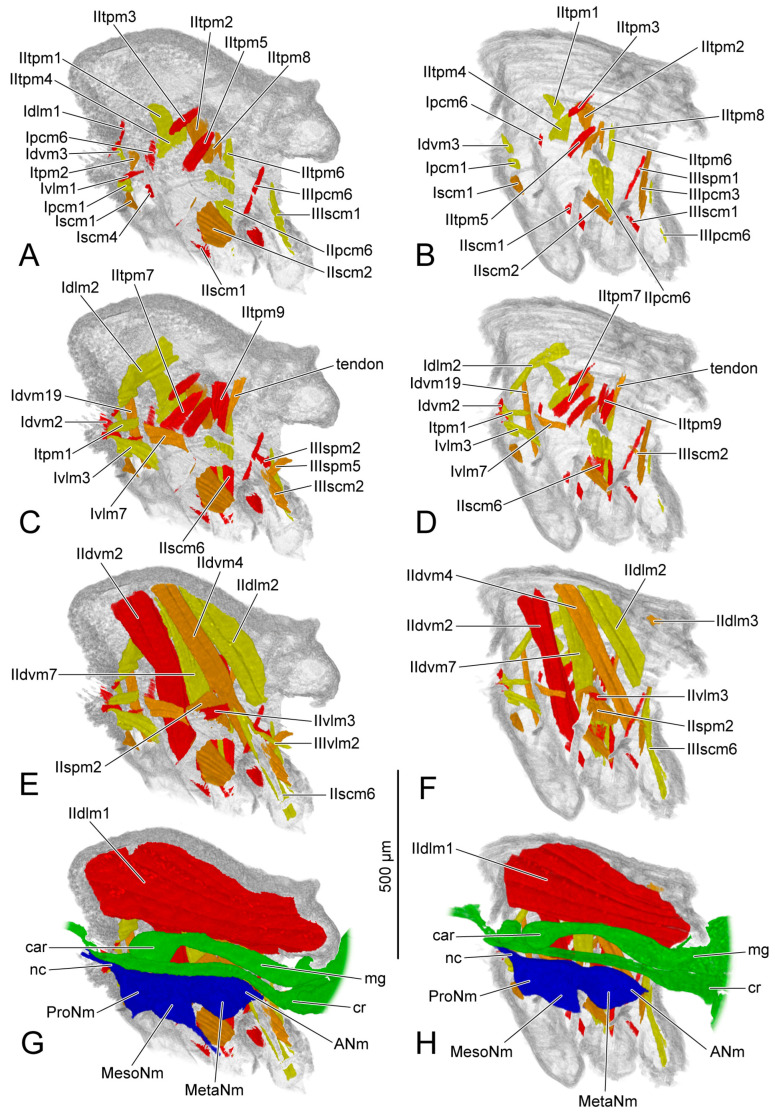
Musculature of *Drosophila melanogaster* in the PD4 sagittal section. Female: (**A**,**C**,**E**,**G**); Male: (**B**,**D**,**F**,**H**). The female IIdlm3 and the male Itpm2, Iscm4, and Ivlm1 are hidden by the skeleton. Abbreviations: ANm–abdominal neuromere; car—cardia; cr—crop; MesoNm–mesothoracic neuromere; MetaNm–metathoracic neuromere; mg—midgut; nc—neck connective.

**Figure 8:**
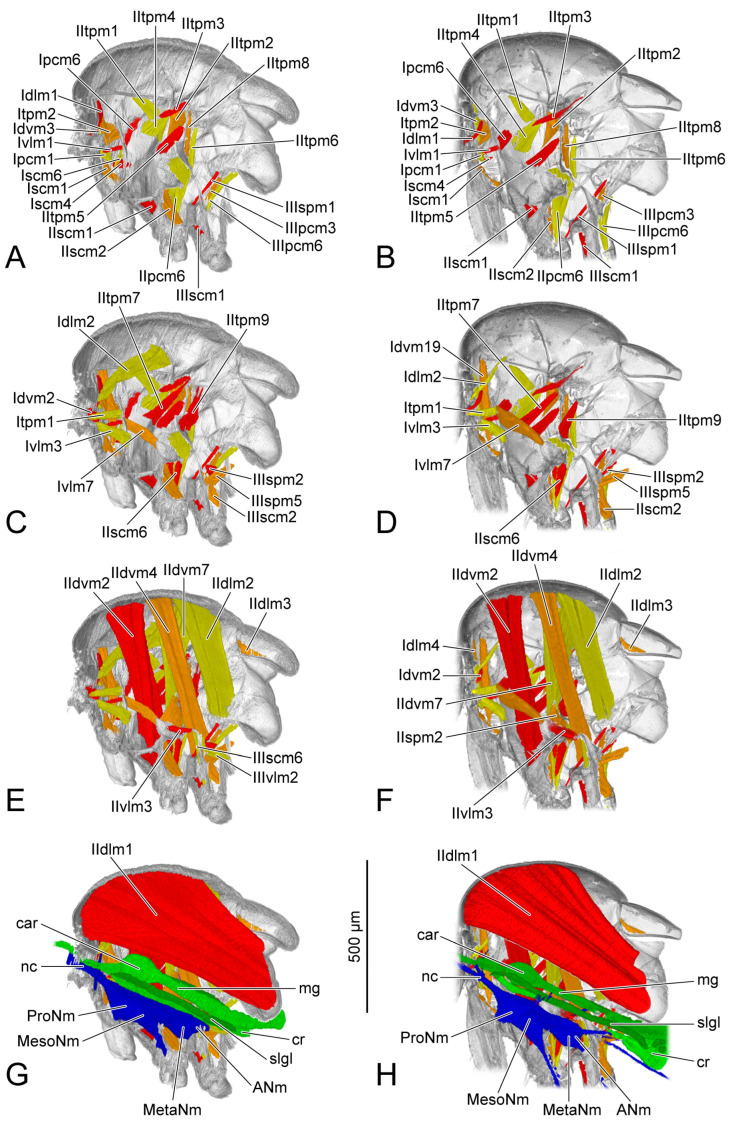
Musculature of *Drosophila melanogaster* in the AD1 sagittal section. Female: (**A**,**C**,**E**,**G**); Male: (**B**,**D**,**F**,**H**). Abbreviations: ANm–abdominal neuromere; car—cardia; cr—crop; MesoNm–mesothoracic neuromere; MetaNm–metathoracic neuromere; mg—midgut; nc—neck connective; slgl—salivary gland.

**Figure 9:**
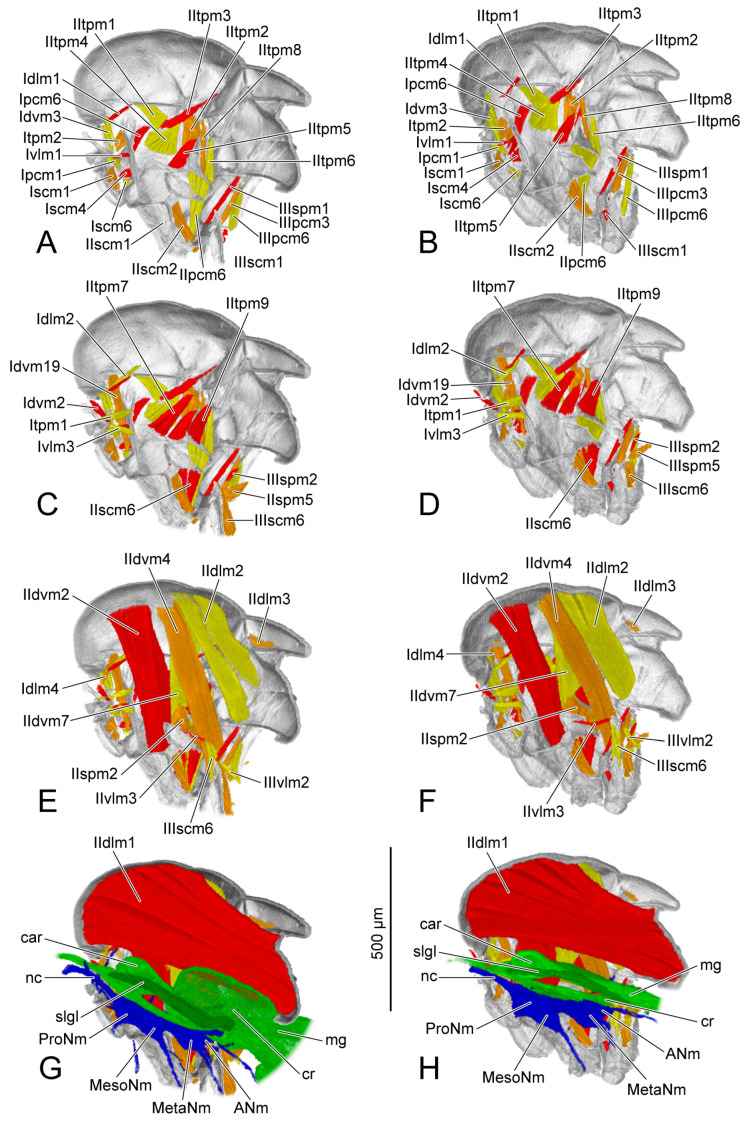
Musculature of *Drosophila melanogaster* in the AD3 sagittal section. Female: (**A**,**C**,**E**,**G**); Male: (**B**,**D**,**F**,**H**). The male IIscm1 is hidden by the skeleton. Abbreviations: ANm–abdominal neuromere; car—cardia; cr—crop; MesoNm–mesothoracic neuromere; MetaNm–metathoracic neuromere; mg—midgut; nc—neck connective; slgl—salivary gland.

The origin and insertion of the muscles on PD3 are recorded. If there are changes in the subsequent periods, they are recorded again. The shape of each muscle is described within every developmental period, with changes in development noted. The presence or absence of muscles in each period was recorded and is presented in Table 4. We also measured the length and the width of each muscle (Figure 5). The absolute length and the absolute width (Table 5 and Table 6) were calculated based on the scale bars in the micro-CT results. For the relative length and the relative width (Table 7 and Table 8), the muscular length and width were compared with the thoracic length, and were calculated according to the following formulas: ML (or MW) × 1000/TL (ML = muscular length; MW = muscular width; TL = thoracic length). We recorded the measurements of the muscles with two bundles in the tables following the order described in the text. For IIdlm1, we measured all bundles together. We compared the female and the male in the same developmental period. For each sex, the comparison was made in developmental order. The 3D visualizations of thoracic musculature are demonstrated in Figure 6, Figure 7, Figure 8 and Figure 9 and Supplementary Videos S1–S8.

#### 3.2.1. Prothoracic Muscles

##### Idlm1 M. prophragma-occipitalis

PD3: Female: O (= origin): dorso-lateral area of pronotum; I (= insertion): dorso-lateral area of occiput. Slender, slightly bent backwards, broad medially and narrowing towards both ends. Male: Absent.

PD4: Female: Slender, bent backwards, broad medially and narrowing towards both ends. Male: Absent.

AD1: Female: Slender, bent backwards, broad medially and narrowing towards both ends. Male: O: dorso-lateral area of pronotum; I: dorso-lateral area of occiput. Slender, bent backwards, broad medially and narrowing towards both ends.

AD3: Female: Slender, straight, broad medially and narrowing towards both ends. Male: Slender, straight, broad medially and narrowing towards both ends.

Developmental changes: Female: The muscle is bent backwards from PD3 to AD1 and straight on AD3. Male: The muscle is bent backwards on AD1 and straight on AD3.

##### Idlm2 M. pronoto-occipitalis

PD3: Female: O: meso-lateral area of mesoscutum; I: dorso-lateral area of occiput. Long, bent lateralward, broad medially and narrowing towards both ends. Male: O: meso-lateral area of mesoscutum; I: dorso-lateral area of occiput. Long, straight, broad medially and narrowing towards both ends.

PD4: Female: Long isosceles triangle, long and broad, bent lateralward, narrowing towards occiput. Male: Long isosceles triangle, long and broad, bent lateralward, narrowing towards occiput.

AD1: Female: Long isosceles triangle, long and broad, bent lateralward, narrowing towards occiput. Male: Approximate long rectangle, long and slender, straight.

AD3: Female: Approximate long rectangle, long and slender, bent lateralward. Male: Long rectangle, straight.

Developmental changes: Female: The original end becomes broader on PD4 and narrower on AD3. The muscle is bent backwards from PD3 to AD1 and straight on AD3. Male: The original end becomes broader on PD4, narrower and bent on AD1. The muscle is straight on PD3, AD1 and AD3 and bent lateralward on PD4.

##### Idlm4 M. cervico-occipitalis dorsalis

PD3: Female: Absent. Male: Absent.

PD4: Female: Absent. Male: Absent.

AD1: Female: Absent. Male: O: dorso-lateral area of pronotum; I: dorso-lateral area of occiput. Long rectangle, short, slightly bent backwards.

AD3: Female: O: dorso-lateral area of pronotum; I: dorso-lateral area of occiput. Long rectangle, short, slightly bent backwards. Male: Long rectangle, short, slightly bent backwards.

Developmental changes: Male: No changes from AD1 to AD3.

##### Idvm2 M. cervico-occipitalis medialis

PD3: Female: O: lateral cervical sclerite; I: dorso-lateral area of occiput. Long rectangle, slender, slightly bent backwards. Male: Absent.

PD4: Female: Long triangle, broad, straight, narrowing towards lateral cervical sclerite. Male: O: lateral cervical sclerite; I: dorso-lateral area of occiput. Slender, straight, broad medially and narrowing towards both ends.

AD1: Female: Long triangle, broad, straight, narrowing towards lateral cervical sclerite. Male: Short and slender, straight, broad medially and narrowing towards both ends.

AD3: Female: Long triangle, broad, slightly bent backwards, narrowing towards occiput. Male: Long triangle, straight, narrowing towards occiput.

Developmental changes: Female: The original end becomes broader on AD3; the insertional end becomes broader on PD4 and narrower on AD3. The muscle is slightly bent backwards on PD3 and AD3 and straight on PD4 and AD1. Male: The original end becomes broader on AD3.

##### Idvm3 M. cervico-occipitalis posterior

PD3: Female: O: lateral cervical sclerite; I: dorso-lateral area of occiput. Short, straight, broad medially and narrowing towards both ends. Male: O: lateral cervical sclerite; I: dorso-lateral area of occiput. Long isosceles triangle, short, straight, narrowing towards occiput.

PD4: Female: Straight, broad medially and narrowing towards both ends. Male: Long isosceles triangle, short, straight, narrowing towards occiput.

AD1: Female: Long isosceles triangle, short, straight, narrowing towards lateral cervical sclerite. Male: Long triangle, straight, narrowing towards lateral cervical sclerite.

AD3: Female: Long isosceles triangle, straight narrowing towards lateral cervical sclerite. Male: Triangle, straight, narrowing towards lateral cervical sclerite.

Developmental changes: Female: The insertional end becomes broader on AD1. Male: The original end becomes narrower on AD1; the insertional end becomes broader on AD1.

##### Idvm19 M. pronoto-trochanteralis

PD3: Female: O: dorso-lateral area of pronotum; I: protrochanter. Long and slender, straight, broad medially and narrowing towards both ends. Male: O: dorso-lateral area of pronotum; I: protrochanter. Long and slender, straight, broad medially and narrowing towards both ends.

PD4: Female: Long, straight, broad medially and narrowing towards both ends. Male: Long and slender, straight, broad medially and narrowing towards both ends.

AD1: Female: Long isosceles triangle, long, straight, narrowing towards protrochanter. Male: Long isosceles triangle, long, straight, narrowing towards protrochanter.

AD3: Female: Long isosceles triangle, long, straight, narrowing towards protrochanter. Male: Long isosceles triangle, long, straight, narrowing towards protrochanter.

Developmental changes: Female: The original end becomes broader on AD1. Male: The original end becomes broader on AD1.

##### Itpm1 M. pleurocrista-occipitalis

PD3: Female: O: ventro-lateral area of occiput; I: antero-dorsal area of profurca. Long triangle, slightly bent upwards, narrowing towards occiput. Male: O: ventro-lateral area of occiput; I: antero-dorsal area of profurca. Straight, broad medially and narrowing towards both ends.

PD4: Female: Long triangle, straight, narrowing towards occiput. Male: Straight, broad medially and narrowing towards both ends.

AD1: Female: Long triangle, straight, narrowing towards occiput. Male: Long isosceles triangle, slender, straight, narrowing towards occiput.

AD3: Female: Straight, broad medially and narrowing towards both ends. Male: Long isosceles triangle, straight, narrowing towards occiput.

Developmental changes: Female: The insertional end becomes broader on PD4 and narrower on AD3. The muscle is bent upwards on PD3 and straight from PD4 to AD3. Male: The insertional end becomes broader on AD1.

##### Itpm2 M. propleuro-occipitalis

PD3: Female: O: lateral cervical sclerite; I: antero-dorsal area of propleural apophysis. Approximate isosceles triangle, short, straight, narrowing towards propleural apophysis. Male: O: lateral cervical sclerite; I: antero-dorsal area of propleural apophysis. Flat triangle, short, straight, narrowing towards propleural apophysis.

PD4: Female: Flat isosceles triangle, short, straight, narrowing towards propleural apophysis. Male: Flat triangle, short, straight, narrowing towards propleural apophysis.

AD1: Female: Flat triangle, broad, straight, narrowing towards propleural apophysis. Male: Flat triangle, broad, straight, narrowing towards propleural apophysis.

AD3: Female: Flat trapezoid, short, straight, narrowing towards lateral cervical sclerite. Male: Flat triangle, broad, straight, narrowing towards propleural apophysis.

Developmental changes: Female: The original end becomes straight on PD4, and the insertional end becomes broader on AD3. Male: No changes from PD3 to AD3.

##### Ipcm1 M. procoxa-cervicalis

PD3: Female: O: antero-lateral margin of procoxal rim; I: lateral cervical sclerite. Long isosceles triangle, short, straight, narrowing towards lateral cervical sclerite. Male: O: antero-lateral margin of procoxal rim; I: lateral cervical sclerite. Long triangle, short, straight, narrowing towards lateral cervical sclerite.

PD4: Female: Long isosceles triangle, short, straight, narrowing towards lateral cervical sclerite. Male: Long isosceles triangle, short, straight, narrowing towards lateral cervical sclerite.

AD1: Female: Long isosceles triangle, short, straight, narrowing towards lateral cervical sclerite. Male: Long isosceles triangle, short, straight, narrowing towards procoxa.

AD3: Female: Long isosceles triangle, short, straight, narrowing towards lateral cervical sclerite. Male: Long rectangle, short.

Developmental changes: Female: No changes from PD3 to AD3. Male: The original end becomes narrower on AD1 and broader on AD3; the insertional end becomes broader on AD1.

##### Ipcm6 M. propleuro-coxalis posterior

PD3: Female: O: anterior area of mesanepisternum; I: lateral margin of procoxal rim. Straight, broad medially and narrowing towards both ends. Male: O: anterior area of mesanepisternum; I: lateral margin of procoxal rim. Straight, broad medially and narrowing towards both ends.

PD4: Female: Straight, broad medially and narrowing towards both ends. Male: Slender, slightly bent downwards, broad medially and narrowing towards both ends.

AD1: Female: Straight, broad medially and narrowing towards both ends. Male: Straight, broad medially and narrowing towards both ends.

AD3: Female: Straight, broad medially and narrowing towards both ends. Male: Long triangle, slightly bent backwards, narrowing towards procoxa.

Developmental changes: Female: No changes from PD3 to AD3. Male: The muscle is straight on PD3 and AD1, slightly bent downwards on PD4 and slightly bent backwards on AD3. The original end becomes broader on AD3.

##### Ivlm1 M. profurca-cervicalis

PD3: Female: O: antero-lateral area of profurcal arm; I: lateral cervical sclerite. Slender, straight, broad medially and narrowing towards both ends. Male: Absent. The muscle is present only in the female.

PD4: Female: Slender, straight, broad medially and narrowing towards both ends. Male: O: antero-lateral area of profurcal arm; I: extends towards lateral cervical sclerite. Short, straight, broad medially and narrowing towards both ends. The muscle is in the process of development.

AD1: Female: Slender, straight, broad medially and narrowing towards both ends. Male: O: antero-lateral area of profurcal arm; I: extends towards lateral cervical sclerite. Slender, straight, broad medially and narrowing towards both ends. The muscle is in the process of development.

AD3: Female: Slender, straight, broad medially and narrowing towards both ends. Male: O: antero-lateral area of profurcal arm; I: lateral cervical sclerite. Slender, straight, broad medially and narrowing towards both ends.

Developmental changes: Female: No changes from PD3 to AD3. Male: The muscle extends forwards until it connects with the lateral cervical sclerite on AD3.

##### Ivlm3 M. profurca-tentorialis

PD3: Female: O: antero-dorsal area of profurca; I: ventro-lateral area of occiput. Slender, straight, broad medially and narrowing towards both ends. Male: O: antero-dorsal area of profurca; I: ventro-lateral area of occiput. Long triangle, slightly bent lateralward, narrowing towards occiput.

PD4: Female: Approximate parallelogram, broad, slightly bent lateralward. Male: Long triangle, straight, narrowing towards occiput.

AD1: Female: Long isosceles triangle, straight, narrowing towards occiput. Male: Long triangle, straight, narrowing towards occiput.

AD3: Female: Slender, straight, broad medially and narrowing towards both ends. Male: Long isosceles triangle, straight, narrowing towards occiput.

Developmental changes: Female: The original end becomes broader on PD4 and narrower on AD3; the insertional end becomes broader on PD4 and narrower on AD1. The muscle is straight on PD3, AD1 and AD3 and lightly bent lateralward on PD4. Male: The muscle is slightly bent lateralward on PD3 and straight from PD4 toAD3.

##### Ivlm7 M. profurca-mesofurcalis

PD3: Female: O: postero-dorsal area of profurca; I: extends to mesofurca. Approximate parallelogram, straight. The muscle is in the process of development. Male: O: postero-dorsal area of profurca; I: extends to mesofurca. Approximate parallelogram, slightly bent lateralward. The muscle is in the process of development.

PD4: Female: O: postero-dorsal area of profurca; I: antero-dorsal area of mesofurca. Parallelogram, straight. Male: O: postero-dorsal area of profurca; I: extends towards mesofurca. Approximate parallelogram, slightly bent lateralward.

AD1: Female: O: postero-dorsal area of profurca; I: antero-dorsal area of mesofurca. Parallelogram, straight. Male: O: postero-dorsal area of profurca; I: antero-dorsal area of mesofurca. Approximate parallelogram, slightly bent lateralward.

AD3: Female: Absent. Male: Absent.

Developmental changes: Female: The muscle extends backwards until it connects with the antero-dorsal area of the mesofurca on PD4. Male: The muscle extends backwards until it connects with the mesofurca on AD1.

##### Iscm1 M. profurca-coxalis anterior

PD3: Female: O: central area of probasisternum; I: antero-lateral margin of procoxal rim. Long triangle, slightly bend backwards, narrowing towards procoxa. Male: O: central area of probasisternum; I: antero-lateral margin of procoxal rim. Long triangle, straight, narrowing towards procoxa.

PD4: Female: O: discrimen of probasisternum; I: antero-lateral margin of procoxal rim. Approximate long triangle, straight, narrowing towards procoxa. Male: O: central area of probasisternum; I: antero-lateral margin of procoxal rim. Long triangle, straight, narrowing towards procoxa.

AD1: Female: Approximate long triangle, straight, narrowing towards procoxa. Male: O: discrimen of probasisternum; I: antero-lateral margin of procoxal rim. Long triangle, straight, narrowing towards procoxa.

AD3: Female: Long triangle, straight, narrowing towards procoxa. Male: Long triangle, straight, narrowing towards procoxa.

Developmental changes: Female: The original site changes from the central area to the discrimen of the probasisternum on PD4. The original end becomes bent on PD4 and straight on AD3. The muscle is slightly bent backwards on PD3 and straight from PD4 to AD3. Male: The original site changes from the central area to the discrimen of the probasisternum on AD1.

##### Iscm4 M. profurca-coxalis lateralis

PD3: Female: O: latero-ventral area of profurcal arm; I: postero-lateral margin of procoxal rim. Two bundles, long triangle, straight, narrowing towards procoxa. Male: O: latero-ventral area of profurcal arm; I: postero-lateral margin of procoxal rim. One bundle, short, straight, broad medially and narrowing towards both ends.

PD4: Female: Two bundles, long triangle, straight, narrowing towards procoxa. Male: One bundle, long triangle, short, straight, narrowing towards profurca.

AD1: Female: One bundle, approximate parallelogram, slightly bent forwards. Male: One bundle, long triangle, short, straight, narrowing towards procoxa.

AD3: Female: One bundle, long triangle, straight, narrowing towards procoxa. Male: Two bundles, long triangular, straight, narrowing towards procoxa.

Developmental changes: Female: Two bundles combine as one bundle on AD1. The insertional end becomes broader on AD1 and narrower on AD3. The muscle is straight on PD3, PD4 and AD3 and slightly bent forwards on AD1. Male: One bundle is divided into two bundles on AD3. The original end becomes broader on PD4 and narrower on AD1; the insertional end becomes broader on AD1.

##### Iscm6 M. profurca-trochanteralis

PD3: Female: Absent. Male: Absent.

PD4: Female: Absent. Male: Absent.

AD1: Female: O: ventro-inner area of the profurcal arm; I: protrochanter. Long triangle, short, straight, narrowing towards protrochanter. Male: Absent.

AD3: Female: Long triangle, short, straight, narrowing towards procoxa. Male: O: ventro-inner area of the profurcal arm; I: protrochanter. Long triangle, short, straight, narrowing towards protrochanter.

Developmental changes: Female: No changes from AD1 to AD3.

#### 3.2.2. Mesothoracic Muscles

##### IIdlm1 M. prophragma-mesophragmalis

PD3: Female: O: anterior to central area of mesoscutum; I: mesophragma. Several bundles fuse together, long and broad. Male: O: anterior to central area of mesoscutum; I: mesophragma. Several bundles fuse together, long and broad.

PD4: Female: Several bundles fuse together, long and broad. Male: Several bundles fuse together, long and broad.

AD1: Female: Several bundles fuse together, long and broad. Male: Several bundles fuse together, long and broad.

AD3: Female: Several bundles fuse together, long and broad. Male: Several bundles fuse together, long and broad.

Developmental changes: Female: No changes from PD3 to AD3. Male: No changes from PD3 to AD3.

##### IIdlm2 M. mesonoto-phragmalis

PD3: Female: O: latero-central area of mesoscutum; I: lateral area of mesophragma. Approximate parallelogram, long and broad, straight. Male: O: latero-central area of mesoscutum; I: lateral area of mesophragma. Approximate parallelogram, long and broad, straight.

PD4: Female: Approximate parallelogram, long and broad, straight. Male: Approximate parallelogram, long and broad, straight.

AD1: Female: Approximate parallelogram, long and broad, straight. Male: Approximate parallelogram, long and broad, straight.

AD3: Female: Two bundles, long and broad, approximate parallelogram, straight. Male: Approximate parallelogram, long and broad, straight.

Developmental changes: Female: The muscle is divided into two bundles on AD3. Male: No changes from PD3 to AD3.

##### IIdlm3

PD3: Female: Absent. Male: Absent.

PD4: Female: O: antero-lateral area of mesoscutellum; I: postero-lateral area of mesoscutellum. Long triangle, straight, narrowing backwards. Male: O: antero-lateral area of mesoscutellum; I: postero-lateral area of mesoscutellum. Long triangle, short, straight, narrowing backwards.

AD1: Female: Long triangle, straight, narrowing backwards. Male: Long triangle, straight, narrowing backwards.

AD3: Female: Long triangle, straight, narrowing backwards. Male: Long triangle, short, straight, narrowing backwards.

Developmental changes: Female: No changes from PD4 to AD3. Male: No changes from PD4 to AD3.

##### IIdvm2 M. mesonoto-trochantinalis anterior

PD3: Female: O: antero-lateral area of mesoscutum; I: ventral area of mesopreepisternum. Approximate parallelogram, long and broad, slightly bent backwards. Male: O: antero-lateral area of mesoscutum; I: ventral area of mesopreepisternum. Approximate parallelogram, long and broad, slightly bent backwards.

PD4: Female: Approximate parallelogram, long and broad, bent backwards. Male: Approximate parallelogram, long and broad, slightly bent backwards.

AD1: Female: Approximate parallelogram, long and broad, straight. Male: Approximate parallelogram, long and broad, straight.

AD3: Female: Approximate parallelogram, long and broad, slightly bent backwards. Male: Approximate parallelogram, long and broad, straight.

Developmental changes: Female: The muscle is bent backwards on PD3, PD4 and AD3 and straight on AD1. Male: The muscle is slightly bent backwards on PD3 and PD4 and straight on AD1 and AD3.

##### IIdvm4 M. mesonoto-coxalis anterior

PD3: Female: O: latero-central area of mesoscutum; I: postero-lateral margin of mesocoxal rim. Long and broad. Approximate parallelogram, long and broad, straight. Male: O: latero-central area of mesoscutum; I: postero-lateral margin of mesocoxal rim. Approximate parallelogram, long and broad, straight.

PD4: Female: Approximate parallelogram, long and broad, straight. Male: Approximate parallelogram, long and broad, straight.

AD1: Female: Approximate parallelogram, long and broad, straight. Male: Approximate parallelogram, long and broad, straight.

AD3: Female: Approximate parallelogram, long and broad, straight. Male: Approximate parallelogram, long and broad, slightly bent backwards.

Developmental changes: Female: No changes from PD3 to AD3. Male: The muscle is straight from PD3 to AD1 and slightly bent backwards on AD3.

##### IIdvm7 M. mesonoto-trochanteralis

PD3: Female: O: lateral area of mesoscutum; I: mesotrochanter. Long triangle, long and broad, slightly bent forwards, narrowing towards mesotrochanter. Male: O: lateral area of mesoscutum; I: mesotrochanter. Approximate long triangle, long and broad, slightly bent forwards, narrowing towards mesotrochanter.

PD4: Female: Long triangle, long and broad, slightly bent forwards, narrowing towards mesotrochanter. Male: Approximate long triangle, long and broad, slightly bent forwards, narrowing towards mesotrochanter.

AD1: Female: Long triangle, long and broad, straight, narrowing towards mesotrochanter. Male: Approximate long triangle, long and broad, straight, narrowing towards mesotrochanter.

AD3: Female: Long triangle, long and broad, curved, narrowing towards mesotrochanter. Male: Approximate long triangle, long and broad, curved, narrowing towards mesotrochanter.

Developmental changes: Female: The muscle is slightly bent forwards on PD3 and PD4, straight on AD1 and curved on AD3. Male: The muscle is slight bent forwards on PD3, PD4, straight on AD1 and curved on AD3.

##### IItpm1 M. prophragma-mesanepisternalis

PD3: Female: O: antero-lateral margin of mesoscutum; I: antero-dorsal area of mesanepisternum. Approximate long triangle, straight, narrowing towards mesoscutum. Male: O: antero-lateral margin of mesoscutum; I: antero-dorsal area of mesanepisternum. Approximate long triangle, straight, narrowing towards mesoscutum.

PD4: Female: Approximate long triangle, straight, narrowing towards mesoscutum. Male: Approximate long triangle, straight, narrowing towards mesoscutum.

AD1: Female: O: antero-lateral margin of mesoscutum; I: mesobasalare. Approximate long triangle, straight, narrowing towards mesoscutum. Male: O: antero-lateral margin of mesoscutum; I: mesobasalare. Approximate parallelogram, straight.

AD3: Female: Approximate parallelogram, straight. Male: Approximate parallelogram, straight.

Developmental changes: Female: The insertional site changes from antero-dorsal area of the mesanepisternum to the mesobasalare on AD1. The original end becomes broader on AD3. Male: The insertional site changes from antero-dorsal area of the mesanepisternum to the mesobasalare on AD1. The original end becomes broader on AD1.

##### IItpm2 M. mesopleura-praealaris

PD3: Female: O: dorsal area of mesopleural apophysis; I: lateral area of mesoscutum. Approximate flat triangle, broad, straight, narrowing towards mesoscutum. Male: Absent.

PD4: Female: Approximate flat triangle, broad, straight, narrowing towards mesoscutum. Male: Flat triangle, broad, straight, narrowing towards mesoscutum.

AD1: Female: Approximate flat triangle, broad, straight, narrowing towards mesoscutum. Male: Long triangle, straight, narrowing towards mesosctum.

AD3: Female: Approximate flat triangle, broad, straight, narrowing towards mesoscutum. Male: Flat triangle, broad, straight, narrowing towards mesoscutum.

Developmental changes: Female: The muscle becomes broader on PD4 and narrower on AD3. Male: The muscle is flat triangle-shaped on PD4 and AD3 and long triangle-shaped on AD1.

##### IItpm3 M. mesonoto-basalaris

PD3: Female: O: lateral area of mesoscutum; I: postero-dorsal area of mesanepisternum. Slightly bent medialward, broad medially and narrowing towards both ends. Male: O: lateral area of mesoscutum; I: postero-dorsal area of mesanepisternum. Short, bent medialward, broad medially and narrowing towards both ends.

PD4: Female: Slightly bent medialward, broad medially and narrowing towards both ends. Male: Slightly bent medialward, broad medially and narrowing towards both ends.

AD1: Female: O: postero-lateral area of mesoscutum; I: mesobasalare. Straight, broad medially, narrowing towards both ends; longer than that on PD4. Male: O: postero-lateral area of mesoscutum; I: mesobasalare. Long and slender, slightly bent medialward, broad medially and narrowing towards both ends.

AD3: Female: Long, straight, broad medially and narrowing towards both ends. Male: Straight, broad medially and narrowing towards both ends.

Developmental changes: Female: The insertional site changes from the postero-dorsal area of the mesanepisternum to the mesobasalare on AD1. The muscle is slight bent medialward on PD3 and PD4 and straight on AD1 and AD3. Male: The insertional position changes from the postero-dorsal area of the mesanepisternum to the mesobasalare on AD1. The muscle is bent medialward from PD3 to AD1 and straight on AD3.

##### IItpm4 M. mesonoto-pleuralis anterior

PD3: Female: O: antero-dorsal area of mesanepisternum; I: lateral margin of mesoscutum. Long isosceles triangle, straight, narrowing towards mesoscutum. Male: O: ventral area of mesanepisternum; I: lateral area of mesoscutum. Long triangle, straight, narrowing towards mesoscutum.

PD4: Female: O: ventral area of mesanepisternum; I: lateral area of mesoscutum. Approximate flat triangle, straight, narrowing towards mesoscutum. Male: Long triangle, straight, narrowing towards mesoscutum.

AD1: Female: Long isosceles triangle, straight, narrowing towards mesoscutum. Male: Approximate long triangle, straight, narrowing towards mesoscutum.

AD3: Female: Long isosceles triangle, straight, narrowing towards mesoscutum. Male: Approximate long triangle, straight, narrowing towards mesoscutum.

Developmental changes: Female: The original site changes from the antero-dorsal area to the ventral area of the mesanepisternum on PD4. The muscle is long isosceles triangle-shaped on PD3, AD1 and AD3 and approximate flat triangle-shaped on PD4. Male: The original end becomes bent on AD1.

##### IItpm5 M. mesonoto-pleuralis medialis

PD3: Female: O: ventral area of mesanepisternum; I: lateral area of mesoscutum. Long triangle, bent medialward, narrowing towards mesoscutum. Male: O: ventral area of mesanepisternum; I: lateral area of mesoscutum. Long triangle, straight, narrowing towards mesoscutum.

PD4: Female: Long triangle, bent medialward, narrowing towards mesoscutum. Male: Long triangle, bent medialward, narrowing towards mesoscutum.

AD1: Female: Two bundles; anterior one: parallelogram, straight; posterior one: long triangle, straight, narrowing towards mesoscutum. Male: Long triangle, straight, narrowing towards mesoscutum.

AD3: Female: Long triangle, straight, narrowing towards mesoscutum. Male: Two bundles; anterior one: long triangle, straight, narrowing towards mesoscutum; posterior one: long triangle, slightly bent medialward, narrowing towards mesoscutum.

Developmental changes: Female: The muscle is divided into two bundles on AD1, and the two bundles combine as one bundle again on AD3. Male: The muscle is divided into two bundles on AD3.

##### IItpm6 M. mesonoto-pleuralis posterior

PD3: Female: O: posterior area of mesopleural apophysis; I: postero-lateral margin of mesoscutum. Slender, straight, broad medially and narrowing towards both ends. Male: O: posterior area of mesopleural apophysis; I: postero-lateral margin of mesoscutum. Long triangle, straight, narrowing towards mesoscutum.

PD4: Female: Long triangle, straight, narrowing towards mesoscutum. Male: Long triangle, slender, straight, narrowing towards mesoscutum.

AD1: Female: Long triangle, slender, straight, narrowing towards mesoscutum. Male: Long triangle, straight, narrowing towards mesoscutum.

AD3: Female: Long triangle, straight, narrowing towards mesoscutum. Male: Long triangle, straight, narrowing towards mesoscutum.

Developmental changes: Female: The original end become broader on PD4. Male: No changes from PD3 to AD3.

##### IItpm7 M. mesanepisterno-axillaris

PD3: Female: O: central area of mesanepisternum; I: lateral margin of mesoscutum. Long triangle, bent medialward, narrowing towards mesoscutum. Male: O: central area of mesanepisternum; I: lateral margin of mesoscutum. Slightly bent medialward, broad medially and narrowing towards both ends.

PD4: Female: Long triangle, slightly bent medialward, narrowing towards mesoscutum. Male: Long triangle, slightly bent medialward, narrowing towards mesoscutum.

AD1: Female: Long isosceles triangle, slightly bent medialward, narrowing towards mesoscutum. Male: Long triangle, straight, narrowing towards mesoscutum.

AD3: Female: Long isosceles triangle, slightly bent medialward, narrowing towards mesoscutum. Male: Long triangle, straight, narrowing towards mesoscutum.

Developmental changes: Female: The muscle is long triangle-shaped on PD3 and PD4 and long isosceles triangle-shaped on AD1 and AD3. The insertional end becomes narrow on PD4. Male: The original end becomes broader on PD4. The muscle is slightly bent medialward on PD3 and PD4 and straight on AD1 and AD3.

##### IItpm8 M. mesepimero-axillaris secundus

PD3: Female: O: posterior area of mesopleural apophysis; I: lateral area of mesoscutum. Straight, broad medially and narrowing towards both ends. Male: O: anterior area of mesopleural apophysis; I: lateral area of mesoscutum. Straight, broad medially and narrowing towards both ends.

PD4: Female: Two bundles; anterior one: long triangle, straight, narrowing towards mesoscutum; posterior one: straight, broad medially and narrowing towards both ends. Male: O: dorsal area of mesopleural apophysis; I: lateral area of mesoscutum. Two bundles; anterior one: approximate long triangle, straight, narrowing towards mesoscutum; posterior one: straight, broad medially and narrowing towards both ends.

AD1: Female: Long triangle, straight, narrowing towards mesoscutum. Male: Two bundles; anterior one: approximate long triangle, straight, narrowing towards mesoscutum; posterior one: straight, broad medially and narrowing towards both ends.

AD3: Female: Long triangle, straight, narrowing towards mesoscutum. Male: Slender, straight, broad medially and narrowing towards both ends.

Developmental changes: Female: The muscle is divided into two bundles on PD4, and the two bundles combine as one bundle again on AD1. The original end becomes broader on AD1. Male: The original site changes from the anterior area to the dorsal area of the mesopleural apophysis from on PD4. The original end becomes straight on AD3. The muscle is divided into two bundles on PD4, and the two bundles combine as one bundle again on AD3.

##### IItpm9 M. mesepimero-axillaris tertius

PD3: Female: O: dorsal area of mesopleural apophysis; I: lateral margin of mesoscutum. Short, straight, broad medially and narrowing towards both ends. Male: O: anterior area of mesopleural apophysis; I: lateral margin of mesoscutum. Straight, broad medially and narrowing towards both ends.

PD4: Female: Two bundles; anterior one: approximate long triangle, straight, narrowing towards mesopleural apophysis; posterior one: approximate long triangle, straight, narrowing towards mesoscutum. Male: O: dorsal area of mesopleural apophysis; I: lateral margin of mesoscutum. Two bundles; anterior one: approximate long triangle, straight, narrowing towards mesopleural apophysis; posterior one: approximate long triangle, straight, narrowing towards mesopleural apophysis.

AD1: Female: Approximate long triangle, straight, narrowing towards mesocutum. Male: Approximate long triangle, straight, narrowing towards mesoscutum.

AD3: Female: Long triangle, straight, narrowing towards mesoscutum. Male: Approximate long triangle, straight, narrowing towards mesoscutum.

Developmental changes: Female: The muscle is divided into two bundles on PD4, and the two bundles combine as one bundle again on AD1. The original end becomes broader on AD1. Male: The original site changes from the anterior area to the posterior-dorsal area of the mesopleural apophysis on PD4. The muscle is divided into two bundles on PD4, and the two bundles combine as one bundle again on AD1. The original end becomes broader on AD1.

##### IIspm2 M. mesofurca-pleuralis

PD3: Female: O: dorso-lateral area of mesofurca; I: dorsal area of mesopleural apophysis. Approximate flat triangle, broad, straight, narrowing towards mesopleural apophysis. Male: O: dorso-lateral area of mesofurca; I: dorsal area of mesopleural apophysis. Approximate flat triangle, broad, straight, narrowing towards mesopleural apophysis.

PD4: Female: Approximate flat triangle, broad, straight, narrowing towards mesopleural apophysis. Male: Approximate flat triangle, broad, straight, narrowing towards mesopleural apophysis.

AD1: Female: Approximate flat triangle, broad, straight, narrowing towards mesopleural apophysis. Male: Approximate flat triangle, broad, straight, narrowing towards mesopleural apophysis. The muscles in both the female and male are the same.

AD3: Female: Approximate flat triangle, broad, straight, narrowing towards mesopleural apophysis. Male: Approximate flat triangle, broad, straight, narrowing towards mesoscutum.

Developmental changes: Female: No changes from PD3 to AD3. Male: No changes from PD3 to AD3.

##### IIpcm6 M. mesopleura-trochanteralis

PD3: Female: O: ventral area of mesopleural apophysis; I: mesotrochanter. Long triangle, long, straight, narrowing towards mesotrochanter. Male: O: ventral area of mesopleural apophysis; I: mesotrochanter. Long triangle, long and broad, straight, narrowing towards mesotrochanter. The muscles in both the female and male are the same.

PD4: Female: Long triangle, long and broad, straight, narrowing towards mesotrochanter. Male: Long triangle, long and broad, straight, narrowing towards mesotrochanter.

AD1: Female: Approximate long triangle, long and broad, straight, narrowing towards mesotrochanter. Male: Approximate long triangle, long and broad, straight, narrowing towards mesotrochanter.

AD3: Female: Approximate long triangle, long and broad, straight, narrowing towards mesotrochanter. Male: Approximate long triangle, long and broad, straight, narrowing towards mesotrochanter.

Developmental changes: Female: The original end becomes bent on AD1. Male: The original end becomes bent on AD1.

##### IIvlm3 M. mesofurca-metafurcalis

PD3: Female: O: postero-dorsal area of mesofurca; I: extends to metafurca. Trapezoid, short, straight. Male: Absent.

PD4: Female: O: postero-dorsal area of mesofurca; I: extends towards metafurca. Approximate parallelogram, straight. Male: O: postero-dorsal area of mesofurca; I: extends towards metafurca. Short, straight, broad medially and narrowing towards both ends.

AD1: Female: O: postero-dorsal area of mesofurca; I: antero-dorsal area of metafurca. Slender, slightly bent medialward, broad medially and narrowing towards both ends. Male: O: postero-dorsal area of mesofurca; I: antero-dorsal area of metafurca. Long triangle, slender, straight, narrowing towards metafurca.

AD3: Female: Slender, straight, broad medially and narrowing towards both ends. Male: Slender, slightly bent medialward, broad medially and narrowing towards both ends.

Developmental changes: Female: The muscle extends backward until connects with the antero-dorsal area of the metafurca on AD1. Both the original end and the insertional end become broader on PD4 and narrower on AD1. The muscle is straight on PD3, PD4 and AD3 and slightly bent medialward on AD1. Male: The muscle extends backward until connects with the antero-dorsal area of the metafurca on AD1. The original end becomes broader on AD1 and narrower on AD3. The muscle is straight on PD4 and AD1 and slightly bent medialward on AD3.

##### IIscm1 M. mesofurca-coxalis anterior

PD3: Female: O: ventral discrimen of mesofurca; I: antero-inner margin of mesocoxal rim. Long triangle, straight, narrowing towards mesocoxa. Male: O: ventral discrimen of mesofurca; I: antero-inner margin of mesocoxal rim. Long triangle, straight, narrowing towards mesocoxa.

PD4: Female: Long triangle, straight, narrowing towards mesocoxa. Male: Long triangle, straight, narrowing towards mesocoxa.

AD1: Female: Long triangle, straight, narrowing towards mesocoxa. Male: Long triangle, straight, narrowing towards mesocoxa.

AD3: Female: Long triangle, straight, narrowing towards mesocoxa. Male: Long triangle, straight, narrowing towards mesocoxa.

Developmental changes: Female: No changes from PD3 to AD3. Male: No changes from PD3 to AD3.

##### IIscm2 M. mesofurca-coxalis posterior

PD3: Female: O: posterior area of mesofurca; I: posterior margin of mesocoxal rim. Approximate parallelogram, straight. Male: O: posterior area of mesofurca; I: posterior margin of mesocoxal rim. Slightly bent downwards, broad medially and narrowing towards both ends.

PD4: Female: Approximate parallelogram, broad, straight. Male: Approximate parallelogram, straight.

AD1: Female: Approximate parallelogram, straight. Male: Slender, straight, broad medially and narrowing towards both ends.

AD3: Female: Approximate parallelogram, straight. Male: Approximate parallelogram, straight.

Developmental changes: Female: No changes from PD3 to AD3. Male: The original end and the insertional end become broader on PD4 and AD3 and narrower on AD1. The muscle is slightly bent downwards on PD3 and straight from PD4 to AD3.

##### IIscm6 M. mesofurca-trochanteralis

PD3: Female: O: ventral area of mesofurca; I: mesotrochanter. Two bundles; anterior one: long triangle, slender, slightly bent forwards, narrowing towards mesotrochaner; posterior one: slender, straight, broad medially and narrowing towards both ends. Male: O: ventral area of mesofurca; I: mesotrochanter. One bundle, approximate parallelogram, slender, straight.

PD4: Female: Two bundles, long triangle, straight, narrowing towards mesotrochanter. Male: Two bundles, long triangle, straight, narrowing towards mesotrochanter.

AD1: Female: Two bundles, long triangle, straight, narrowing towards mesotrochanter. Male: Two bundles, long triangle, straight, narrowing towards mesotrochanter.

AD3: Female: Two bundles, long triangle, straight, narrowing towards mesotrochanter. Male: Two bundles, long triangle, straight, narrowing towards mesotrochanter.

Developmental changes: Female: The original end of the posterior bundle becomes broader on PD4. The muscle is slightly bent forwards on PD3 and straight from PD4 to AD3. Male: The muscle is divided into two bundles on PD4.

##### Tendon

PD3: Female: O: posterior area of metapleural apophysis; I: postero-lateral area of mesoscutum. Approximate parallelogram, straight. Male: O: posterior area of metapleural apophysis; I: postero-lateral area of mesoscutum. Short, straight, broad medially and narrowing towards both ends.

PD4: Female: O: postero-dorsal area of metapleural apophysis; I: postero-lateral area of mesoscutum. Long triangle, straight, narrowing towards metapleural apophysis. Male: O: postero-dorsal area of metafurca; I: postero-lateral area of mesoscutum. Long and slender, curved, broad medially and narrowing towards both ends.

AD1: Female: Absent. Male: Absent.

AD3: Female: Absent. Male: Absent.

Developmental changes: Female: The original end becomes narrower on PD4. Male: The tendon is straight on PD3 and curved on PD4.

#### 3.2.3. Metathoracic Muscles

##### IIIspm1 M. metapleural-sternalis

PD3: Female: O: dorsal area of metapleuron; I: antero-lateral margin of metacoxal rim. Slender, straight, broad medially and narrowing towards both ends. Male: O: central area of metapleuron; I: lateral margin of metacoxal rim. Short, straight, broad medially and narrowing towards both ends.

PD4: Female: Approximate parallelogram, slender, straight. Male: O: dorsal area of metapleuron; I: antero-lateral area of metacoxal rim. Long and slender, straight, broad medially and narrowing towards both ends.

AD1: Female: O: postero-dorsal area of metapleuron; I: antero-lateral margin of metacoxal rim. Approximate parallelogram, slender, straight. Male: O: postero-dorsal area of metapleuron; I: antero-lateral margin of metacoxal rim. Long and slender, slightly bent lateralward, broad medially and narrowing towards both ends.

AD3: Female: Approximate parallelogram, long and slender, straight. Male: Long and slender, straight, broad medially and narrowing towards both ends.

Developmental changes: Female: The original site changes from the central area to the postero-dorsal area of the metapleuron on PD4. Both the original end and the insertional end become broader on PD4. Male: The original site changes from the central area to the dorsal area of the metapleuron on PD4, and to the postero-dorsal area of the metapleuron on AD1. The insertional site changes from the lateral margin of the metacoxal rim to the lateral area of the metabasisternum on PD4. The muscle is straight on PD3, PD4 and AD3 and slightly bent lateralward on AD1.

##### IIIspm2 M. metafurca-pleuralis

PD3: Female: Absent. Male: Absent.

PD4: Female: O: dorso-lateral area of metafurca; I: metapleural apophysis. Approximate parallelogram, broad, straight. Male: Absent.

AD1: Female: Approximate parallelogram, short, straight. Male: O: dorso-lateral area of metafurca; I: metapleural apophysis. Short and slender, bent backwards, broad medially and narrowing towards both ends.

AD3: Female: Short and slender, straight, broad medially and narrowing towards both ends. Male: Long triangle, short, slightly bent forwards, narrowing towards metapleural apophysis.

Developmental changes: Female: Both the original end and insertional end becomes narrower on AD3. Male: The original end becomes broader on AD3. The muscle is bent backwards on AD1 and slightly bent forwards on AD3.

##### IIIspm5 M. metafurca-intersegmentalis posterior

PD3: Female: Absent. Male: Absent.

PD4: Female: O: postero-dorsal area of metafurca; I: intersegmental sclerite between metathorax and abdominal pleura. Approximate parallelogram, slightly bent upwards. Male: Absent.

AD1: Female: Approximate parallelogram, slightly bent upwards. Male: O: postero-dorsal area of metafurca; I: intersegmental sclerite between metathorax and abdominal pleura. Approximate parallelogram, straight.

AD3: Female: Approximate parallelogram, straight. Male: Approximate parallelogram, straight.

Developmental changes: Female: The muscle is slightly bent upwards on PD4 and AD1 and straight on AD3. Male: No changes from AD1 to AD3.

##### IIpcm3 M. metanepisterno-coxalis anterior

PD3: Female: Absent. Male: O: postero-dorsal area of metapleuron; I: antero-lateral margin of metacoxal rim. Long and slender, slightly bent lateralward, broad medially and narrowing towards both ends.

PD4: Female: Absent. Male: Long and slender, straight, broad medially and narrowing towards both ends.

AD1: Female: O: postero-dorsal area of metapleuron; I: antero-lateral margin of metacoxal rim. Straight, broad medially and narrowing towards both ends. Male: Slender; straight, broad medially and narrowing towards both ends.

AD3: Female: Slender, straight, broad medially and narrowing towards both ends. Male: Slender, broad medially and narrowing towards both ends.

Developmental changes: Female: No changes from AD1 to AD3. Male: The muscle is slightly bent lateralward on PD3 and straight from PD4 to AD3.

##### IIIpcm6 M. metapleural-trochanteralis

PD3: Female: O: postero-dorsal area of metapleuron; I: metatrochanter. Long and slender, bent forwards, broad medially and narrowing towards both ends. Male: O: postero-dorsal area of metapleuron; I: metatrochanter. Straight, broad medially and narrowing towards both ends.

PD4: Female: Long and slender, bent forwards, broad medially and narrowing towards both ends. Male: Long and slender, slightly bent backwards, broad medially and narrowing towards both ends.

AD1: Female: Slender, bent forwards, broad medially and narrowing towards both ends. Male: Long and slender, bent forwards, broad medially and narrowing towards both ends.

AD3: Female: Straight, broad medially and narrowing towards both ends. Male: Long triangle, long, straight, narrowing towards metatrochanter.

Developmental changes: Female: The muscle is bent forwards from PD3 to AD1 and straight on AD3. Male: The original end becomes broader on AD3. The muscle is straight on PD3 and AD3, bent backwards on PD4 and bent forwards on AD1.

##### IIIvlm2 M. metafurca-abdominosternalis

PD3: Female: Absent. Male: Absent.

PD4: Female: O: postero-dorsal area of metafurca; I: abdominal sternite. Long triangle, slender, slightly bent lateralward, narrowing towards abdomen. Male: Absent.

AD1: Female: Long triangle, slender, straight, narrowing towards abdomen. Male: Absent.

AD3: Female: Long triangle, slender, straight, narrowing towards abdomen. Male: O: postero-dorsal area of metafurca; I: abdominal sternite. Long triangle, straight, narrowing towards abdomen.

Developmental changes: Female: The muscle is slightly bent lateralward on PD4 and straight on AD1 and AD3. Male: The muscle is present only on AD3.

##### IIIscm1 M. metafurca-coxalis anterior

PD3: Female: Absent. Male: Absent.

PD4: Female: O: postero-ventral area of metafurca; I: antero-inner margin of metacoxal rim. Approximate parallelogram, broad, straight. Male: O: postero-ventral area of metafurca; I: antero-inner margin of metacoxal rim. Approximate parallelogram, straight.

AD1: Female: Approximate parallelogram, broad, straight. Male: Approximate parallelogram, straight.

AD3: Female: Straight, broad medially and narrowing towards both ends. Male: Approximate parallelogram, straight.

Developmental changes: Female: Both the original end and insertional end become narrower on AD3. Male: No changes from PD4 to AD3.

##### IIIscm2 M. metafurca-coxalis posterior

PD3: Female: O: posterior area of metafurca; I: postero-inner margin of metacoxal rim. Approximate parallelogram, straight. Male: O: posterior area of metafurca; I: postero-inner margin of metacoxal rim. Straight, broad medially and narrowing towards both ends.

PD4: Female: Approximate parallelogram, broad, slightly bent downwards. Male: Short and slender, straight, broad medially and narrowing towards both ends.

AD1: Female: Approximate parallelogram, straight. Male: Long triangle, straight, narrowing towards metafurca.

AD3: Female: Approximate parallelogram, straight. Male: Approximate parallelogram, straight.

Developmental changes: Female: The muscle is straight on PD3, AD1 and AD3 and slightly bent downwards on PD4. Male: The insertional end becomes narrower on AD1 and broader on AD3.

##### IIIscm6 M. metafurca-trochanteralis

PD3: Female: O: postero-dorsal area of metafurca; I: metatrochanter. Long, slightly bent backwards, broad medially and narrowing towards both ends. Male: O: postero-dorsal area of metafurca; I: metatrochanter. Long, broad medially and narrowing towards both ends.

PD4: Female: Approximate parallelogram, long, straight. Male: Long, slightly bent forwards, broad medially and narrowing towards both ends.

AD1: Female: Approximate parallelogram, long, straight. Male: Long, straight, broad medially and narrowing towards both ends.

AD3: Female: Approximate parallelogram, long, straight. Male: Approximate parallelogram, long, straight.

Developmental changes: Female: Both the original end and the insertional end become broader on PD4. The muscle is slightly bent backwards on PD3 and straight from PD4 to AD3. Male: Both the original end and insertional end become broader on AD3. The muscle is straight on PD3, AD1 and AD3 and slightly bent forwards on PD4.

### 3.3. Ventral Nerve Cord

PD3 (vnc: Figure 1A,B; blue structure: Figure 6G,H): The ventral nerve cord is roughly divided into three sections corresponding to the three thoracic segments: prothoracic neuromere (ProNm: Figure 6G,H), mesothoracic neuromere (MesoNm: Figure 6G,H) and metathoracic neuromere (MetaNm: Figure 6G,H). The small abdominal neuromere (ANm: Figure 6G,H) is located behind the metathoracic neuromere. The female ventral nerve cord has formed the anterior neck connective (nc: Figure 6G) and a posterior process extending towards the abdomen.

PD4 (vnc: Figure 1D; blue structure: Figure 7G,H): Anteriorly, the neck connective (nc: Figure 7G,H) extends into the head. The female prothoracic (ProNm: Figure 7G) and mesothoracic neuromeres (MesoNm: Figure 7G) each have a pair of ventral processes extending towards the legs. The process of the mesothoracic neuromere is much longer.

AD1 (blue structure: Figure 8G,H): The ventral nerve cord develops faster in the male than in the female. Compared with the female, the male has more processes including a pair of dorsal processes extending into the dorsal area of the thorax, and a pair of ventral processes of metathoracic neuromere (MetaNm: Figure 8H) extending into the hindlegs.

AD3 (blue structure: Figure 9G,H): Both male and female ventral nerve cords are well developed. The ventral nerve cord has a furcate neck connective (nc: Figure 9G,H) connecting with the brain, a posterior process entering the abdomen, several dorsal processes of meso- and metathoracic neuromeres, and three pairs of ventral processes, respectively, extending into the pro-, mid- and hindlegs.

### 3.4. Digestive System

The section of digestive system located in the thorax is the most anterior part of the midgut. Before that, the crop (cr: Figure 6G,H, Figure 7G,H, Figure 8G,H and Figure 9G,H) branches out from the foregut. Anteriorly, the midgut has a ball-shaped cardia (car: Figure 6G,H, Figure 7G,H, Figure 8G,H and Figure 9G,H). The volume of the crop depends on food intake.

PD3 (green structure: Figure 6G,H): The part posterior to the cardia is bent upwards in the female. The volume of the crop (cr: Figure 6G) in the female is larger than that in the male (cr: Figure 6H).

PD4 (green structure: Figure 7G,H): The midguts in both female and male thoraxes are same. The volumes of posterior part of the midgut and the crops decrease compared with those on PD3.

AD1 (green structure: Figure 8G,H): Both the female and the male have the midguts narrower than that on PD4. The female midgut is slightly broader than that in the male. The salivary gland (slgl: Figure 8G,H) on each side of the midgut extending to the anterior area of abdomen can be identified from the micro-CT results.

AD3 (green structure: Figure 9D,H): The female crop (cr: Figure 9G) becomes much broader than that on AD1. The male midgut shows no change compared with that on AD1.

## 4. Discussion

### 4.1. Observation of the Anatomical Structure of D. melanogaster Based on Improved Morphological Techniques

Robertson [14] embedded samples in paraffin to make a series of histological sections with the assistance of fixatives and stains, in order to observe anatomical structures such as the hypodermis, imaginal discs and muscular fibers in clipping planes. Bainbridge and Bownes [15] used a dissecting microscope with an attached camera to observe anatomical structures with the help of scattered light inside wetted pupae. They were able to observe changes to the exoskeleton during metamorphosis, as well as the Malphighian tubules, trunk trachea, and floated bubble. The schematic figures depicted by Hartenstein [17] show the central nervous system, intestinal tract, and somatic musculature, with general outlines. Similarly, Zlokar [18], Ferris [19], and Miller [20] illustrated external and internal schematic structures via hand-drawings made using light microscopes. These hand-drawings require the authors have superb drawing skills, which necessitates extensive training. Combined with an optimized workflow, computer-based 3D reconstructions using micro-CT image stacks greatly facilitate analyses of the anatomical structures of small animals [26]. In this study, we use these advanced techniques to exhibit a highly simulated organ system, including the skeleton, muscles, nervous ganglion, and gut. The fibers on some stronger muscles are even visible.

### 4.2. Transformations during Development

The basic structure of the endoskeleton has been formed on PD3. The internal ridges and apophysis continue to develop until the adult stage; this might be the reason for the changes in the original sites of some muscles. The discrimen of the probasisternum appears, respectively, in the female on PD4 and in the male on AD1, as the new original site of Iscm1 (Figure 3B,C). The male mesopleural apophysis rotates forwards from PD3 to PD4, which possibly causes changes in the original sites of the male IItpm8 and 9 from the anterior area to the dorsal area of the mesopelural apophysis (Figure 3D–G). Otherwise, the original sites, the female Iitpm4 and the female and the male muscle IIIspm1, change in the pleuron; this may be influenced by the enlargement of the thoracic sclerites.

The muscles change in many ways during development, rather than simply growing longer and broader. Almost all muscles become shorter or narrower either in absolute length and width or relative to body size after a certain period of development (Table 5, Table 6, Table 7 and Table 8). It should be noted that the results of measuring the muscular length and width are affected by factors such as the quality of 3D reconstruction and changes in muscular shape. Many muscles undergo changes in their shapes, as shown in Figure 5. Some muscles change from straight to bent or curved, while some others do the opposite. Some muscles change the number of bundles. The division of one bundle into two bundles occurs in the female IIdlm2, in the male Iscm4 and IIscm6, and in both female and male IItpm5, 8 and 9. On the contrary, the two bundles combine as one bundle in the female Iscm4 and IItpm5, and in both female and male IItpm8 and 9. Ivlm1, Ivlm7, and IIvlm3 demonstrate the gradual extension of the muscular formation from the original site to the insertional site [27]. The free end of the muscle might be fixed by the other soft tissues in the stroma, instead of scaffolds formed by larval fibers [17,28]. In holometabolous insects, muscles have well-characterized contact with other tissues during development [29]. The absence of the mesothoracic tendon and the muscle Ivlm7, respectively, on AD1 and AD3, is probably due to increased sclerization in the adult stage. In apterygote hexapods, the separated anterior and posterior tentorial arms are connected by muscles, whereas Pterygota, with a more complete skeletal structure, do not have these muscles [30].

Nearly all flight-related muscles, especially the dorso-longitudinal muscles, the dorso-ventral muscles, and the tergo-pleural muscles in the mesothorax, appeared on PD3, except the IItpm2 in the male. No matter how the muscular size and shape change, the weight of the flight muscles may become greater after adult eclosion, and the mitochondrial content also increases to that of the cricket *Teleogryllus oceanicus* [31]. The appearance of indirect flight muscles in the early pupal stage has also been recorded in the development of *Calliphora vicina* [32] and *Megachile rotundata* [33]. A study of hover flight has estimated that muscular efficiency is quite low, based on a comparison of the mechanical power output with the metabolic power input of the flight muscles [34]. The reduced body size of flies was also pointed out to impose steep constraints on their flight ability [35]; thus, their wings must flap faster to generate sufficient forces to stay aloft. To meet aerodynamic power requirements, flight muscles need a large number of myofibers containing abundant mitochondria for metabolism. Therefore, an earlier appearance gives the flight muscles more time to enhance their metabolism and energy output. In contrast to the flight muscles, some muscles that connect the legs or fix the structure of the body are still absent on PD3. Some muscles do not appear until the third day after the emergence, such as Idlm4 in the female and Iscm6 and IIIvlm2 in the male, thereby differing from the thoracic muscles of *Chrysopa pallens* [27], in which muscular development was confined to the pupal stage.

The muscles Idlm1, Idvm2, Ivlm1, Iscm6, IItpm2, IIvlm3, and IIspm2 and 5 appear earlier in the female, whereas the muscles Idlm4 and IIIpcm3 are initially present in the male. In the same developmental period, the homologous muscles in the female usually have longer and broader absolute lengths and widths than those in the male (Table 5 and Table 6). Correspondingly, the female also has a longer thoracic length (Table 3). Some muscular shapes, origins and insertions in the female and the male are different. The sexual differences also refer to the development of the nervous system and digestive system. After the eclosion, the male ventral nerve cord develops faster than that in the female. The differences in the crop volumes on PD3 cannot be explained at the moment. On AD3, the female has a significantly larger crop than that in the male. Currently, we can only speculate the female starts eating earlier than the male, in order to store energy for oviposition. It has been recorded that females exhibit complex foraging patterns when feeding on food and laying eggs, depending on their own nutritional state [36].

### 4.3. Serial Homology during Metamorphosis

Wipfler et al. [22] described the skeletomuscular system of the third instar larva of *D. melanogaster*. The larva has a legless thorax covered by a thin, weakly sclerotized and transparent cuticle. The pseudocephalon retracts in the prothorax, and the meso- and metathorax are nearly the same. Only the longitudinal muscles and the dorso-ventral muscles are present in the thorax of the larva. In contrast, the thorax of the adult has a strongly sclerotized skeleton, with huge differences compared with the larva. The mesothorax is significantly larger than the pro- and metathorax. Apart from the longitudinal muscles and dorso-ventral muscles, the thorax has the tergo-pleural muscles, sterno-pleural muscles, pleuro-coxal muscles and sterno-coxal muscles. Intuitively, we consider that the segmental longitudinal muscles and dorso-ventral muscles of each thoracic segment in the larva are, respectively, homologous with the corresponding longitudinal muscles and dorso-ventral muscles of the adult, depending on their attachment positions. However, homology is difficult to determine due to the complexity of the muscle formation process. The dorsal longitudinal muscles of the larva of *Drosophila* have been found persisting into metamorphosis and serving as scaffolds for the formation of the dorsal longitudinal flight muscles of the adult [28]. During metamorphosis, the muscular system is destroyed and replaced by an entirely new set of muscles in the adult [14,37]. Although some of the muscles in the adult share similar origins and insertions with those in the larva, it is likely that they are different muscles and have no homologous relationship.

If we consider that the longitudinal muscles and the dorso-ventral muscles of the larva are homologous with those in the adult, the functions of most muscles change. In the larva, longitudinal muscles are proposed as the power source of peristalsis. In the adult, the mesothoracic dorsal longitudinal muscles (IIdlm1 and 2), which are considered homologous with the mesothoracic dorsal segmental longitudinal muscles of the larva and are the principal wing depressors and levators, provide power for flight [38]. The prothoracic dorsal longitudinal muscles Idlm1, 2, and 4 in the adult might be homologous with the prothoracic dorsal segmental longitudinal muscles of the lava, and control the head activity. The ventral longitudinal muscles Ivlm1, 3 and 7 in the prothorax, IIvlm3 in the mesothorax and IIIvlm2 in the metathorax may, respectively, be homologous with the prothoracic ventral segmental longitudinal muscles, the mesothoracic ventral segmental longitudinal muscles, and the metathoracic intersegmental muscles M20 or 21, which connect the ventral walls of the metathorax and the abdominal segment in the larva. These ventral longitudinal muscles in the adult no longer provide direct power for body movement. The thoracic dorso-ventral muscles in the larva might only support the body structure, except the intersegmental muscle M12. M12, which connects the dorsal wall of prothorax and the ventral wall of pseudocephalon, controls the activity of the pseudocephalon. In the adult, this muscle is probably replaced by the prothoracic dorso-longitudinal muscles Idlm1, 2, and 4 and the dorso-ventral muscles Idvm2 and 3 that connect the occiput and the pronotum, mesoscutum, and lateral cervical sclerite. With the appearance of the wings and legs, the other dorso-ventral muscles in the adult serve more alternative functions. The mesothoracic muscles IIdvm2, 4, and 7 are the indirect wing levators in flying insects [38]. The prothoracic muscle Idvm19, inserting the protrochanter, is obviously involved in walking and jumping.

To achieve complex flight movements, the adult also develops direct flight muscles, specifically the tergo-pleural muscles IItpm1–9, which attach to the wing base sclerites. Those muscles in the pterothorax function as the wing pronator or supinator [38]. Heming [39] classified the muscular transformation and metamorphosis based on whether they only exist in either the larva or the adult, and whether their functions change. Apparently, changes in the thoracic skeleto-muscular system and its related functions from the larva stage to the adult stage are reflective of their respective behaviors and habitats [40]. The larvae inhabit and feed on fermenting fruit. The adults need to perform courtship, mating, and oviposition. It is inferred that the adults also roam in search of new food sources.

The entire central nervous system in the larva is located in the metathorax and the first abdominal segment, and lacks a defined segmental ventral nervous cord [22]. In the adult, the thoracic ventral nerve cord is clearly divided into three neuromeres according to the three thoracic segments and a small posterior abdominal neuromere. Dorsally, the tectulum of the ventral nerve cord undertakes some processes to primarily control the locomotion of wings. Ventrally, the leg neuropil of each thoracic neuromere has a pair of processes extending to the leg. The adult has more commissures per neuromere than the larva, as the larval commissures split into additional pathways during metamorphosis due to the expansion and extension of the neuropil [25,41]. The ventral nerve cord in the adult coordinates not only wing and leg muscles but also abdominal movement, and receives a wide range of mostly mechano- and chemosensory input [25,42,43].

Both the larva and adult have cardia in the anterior area of the midgut. The diameters of the midgut and the salivary gland from the thoracic ventral area to the abdomen gradually increase. The salivary gland in the larva is larger than that in the adult. The general similarity in the midguts might suggest that the adult and larva have similar feeding habits, such as fermenting fruits [40]. Hartenstein [17] showed a strong reduction in the size of the pupal midgut compared with that in the larva. However, based on our comparison, the midgut in the pupa is as broad as or even broader than that in the larva. The differences between the results of Hartenstein [17] and ours are probably due to whether or not the larva ate before pupation.

## 5. Conclusions

This study shows the morphological transformation of the thorax of *D. melanogaster* from the third day of the pupal stage to the third day after emergence. The endoskeleton, musculature, ventral nerve cord, and digestive system are exhibited and described in detail using micro-CT and 3D visualization. The original sites of some muscles change as the endoskeleton develops. Changes in muscular size during metamorphosis not only include becoming longer or broader, but also becoming shorter or narrower, in both absolute and relative terms. The muscular shape during development might be different. The number of muscular bundles can also increase or decrease. The free ends of the growing muscles might be anchored by the soft tissues in the stroma. The increased sclerization of the adult probably causes the loss of some muscles and tendons. Almost all the flight muscles were present on PD3, and completed their development on AD1, probably because the flight muscles need numerous myofibers with enough mitochondria to meet aerodynamic power requirements. There are some differences between the male and the female muscles, ventral nerve cord, and digestive system in the same developmental period. We propose most thoracic muscles have changed their functions from supporting peristalsis in larvae to supporting flight and walking in adults, although the serial homology during metamorphosis might be inconclusive. The ventral nerve cord in the adult has obvious neuromeres with more commissures to control the more complicated locomotion and receive multimodal sensory input. The similarity in the endodermal digestive systems of larvae and adults might reflect their similar feeding habits. In the future, we will continue to demonstrate the anatomical structures of other body segments and other stages during metamorphosis, including eggs, larvae, and the early pupal stage, using a wider variety of morphological techniques.

## Figures and Tables

**Table 1:** Formulae of growth medium and preservative.

Growth Mediun		Preservative	
Materials	Dosage	Materials	Dosage
Agar	33.6 g	95% Ethanol	1 L
Sugar	94.86 g	Butylparaben	500 g
Glucose	189.6 g		
Corn Flour	233.1 g		
Yeast	90 g		
Water	6 L		

**Table 2:** Parameters of micro-CT scanning and 3D reconstruction.

Parameters	PD3 Female	PD4 Male	PD4 Female	PD4 Male	AD1 Female	AD1 Male	AD3 Female	AD1 Male
Voltage	60 kV	60 kV	60 kV	60 kV	50 kV	50 kV	60 kV	60 kV
Current	130 µA	130 µA	130 µA	130 µA	120 µA	120 µA	130 µA	130 µA
Pixel Size	1.8554 µm	1.9049 µm	1.6421 µm	1.9843 µm	2.8783 µm	2.8783 µm	2.8783 µm	2.8783 µm
Voxel Size	6.387 µm^3^	6.912 µm^3^	4.428 µm^3^	7.813 µm^3^	23.85 µm^3^	23.85 µm^3^	23.85 µm^3^	23.85 µm^3^
Exposure Time	1.5000 s	2.0000 s	2.2000 s	1.5000 s	3.0000 s	2.5000 s	3.0000 s	3.0000 s
Image Size	1012 × 1012							
Optical Magnification	3.9687: “4×”							

**Table 3:** Thoracic length of *Drosophila melanogaster* (from the anterior margin of the mesoscutum to the posterior margin of the mesopostnotum).

	PD3	PD4	AD1	AD3
Female	635.4 µm	831.1 µm	989.6 µm	1012 µm
Male	500.0 µm	657.3 µm	714.3 µm	769.2 µm

**Table 4:** The thoracic muscles in each period. Present is denoted with “+” with green color, and absent is denoted with “-” with orange color.

	PD3		PD4		AD1		AD3	
Muscle	Female	Male	Female	Male	Female	Male	Female	Male
Prothorax								
Idlm1	+	-	+	-	+	+	+	+
Idlm2	+	+	+	+	+	+	+	+
Idlm4	-	-	-	-	-	+	+	+
Idvm2	+	-	+	+	+	+	+	+
Idvm3	+	+	+	+	+	+	+	+
Idvm19	+	+	+	+	+	+	+	+
Itpm1	+	+	+	+	+	+	+	+
Itpm2	+	+	+	+	+	+	+	+
Ipcm1	+	+	+	+	+	+	+	+
Ipcm6	+	+	+	+	+	+	+	+
Ivlm1	+	-	+	+	+	+	+	+
Ivlm3	+	+	+	+	+	+	+	+
Ivlm7	+	+	+	+	+	+	-	-
Iscm1	+	+	+	+	+	+	+	+
Iscm4	+	+	+	+	+	+	+	+
Iscm6	-	-	-	-	+	-	+	+
Mesothorax								
IIdlm1	+	+	+	+	+	+	+	+
IIdlm2	+	+	+	+	+	+	+	+
IIdlm3	-	-	+	+	+	+	+	+
IIdvm2	+	+	+	+	+	+	+	+
IIdvm4	+	+	+	+	+	+	+	+
IIdvm7	+	+	+	+	+	+	+	+
IItpm1	+	+	+	+	+	+	+	+
IItpm2	+	-	+	+	+	+	+	+
IItpm3	+	+	+	+	+	+	+	+
IItpm4	+	+	+	+	+	+	+	+
IItpm5	+	+	+	+	+	+	+	+
IItpm6	+	+	+	+	+	+	+	+
IItpm7	+	+	+	+	+	+	+	+
IItpm8	+	+	+	+	+	+	+	+
IItpm9	+	+	+	+	+	+	+	+
IIspm2	+	+	+	+	+	+	+	+
IIpcm6	+	+	+	+	+	+	+	+
IIvlm3	+	-	+	+	+	+	+	+
IIscm1	+	+	+	+	+	+	+	+
IIscm2	+	+	+	+	+	+	+	+
IIscm6	+	+	+	+	+	+	+	+
tendon	+	+	+	+	-	-	-	-
Metathorax								
IIIspm1	+	+	+	+	+	+	+	+
IIIspm2	-	-	+	-	+	+	+	+
IIIspm5	-	-	+	-	+	+	+	+
IIIpcm3	-	+	-	+	+	+	+	+
IIIpcm6	+	+	+	+	+	+	+	+
IIIvlm2	-	-	+	-	+	-	+	+
IIIscm1	-	-	+	+	+	+	+	+
IIIscm2	+	+	+	+	+	+	+	+
IIIscm6	+	+	+	+	+	+	+	+

**Table 5:** The absolute lengths (µm) of thoracic muscles in each period. Within the same sex, the absolute length longer than that in the previous developmental period is denoted in blue, while the shorter length is denoted in yellow. In the same period, the longer absolute length is denoted in bold. Absent is denoted with “-”.

	PD3		PD4		AD1		AD3	
Muscle	Female	Male	Female	Male	Female	Male	Female	Male
Prothorax								
Idlm1	83.33	-	167.3	-	**175.2**	85.71	**146.0**	115.7
Idlm2	**196.7**	129.6	**265.9**	194.7	**342.0**	195.8	**265.4**	131.1
Idlm4	-	-	-	-	-	85.71	**99.54**	62.68
Idvm2	92.92	-	**122.1**	70.12	**104.5**	52.91	**159.3**	120.8
Idvm3	50.83	**58.15**	**126.4**	61.34	55.16	**123.3**	120.8	**155.0**
Idvm19	**305.0**	197.4	**312.8**	209.3	**319.9**	233.3	**333.8**	284.9
Itpm1	**115.8**	88.89	**114.4**	83.74	**157.0**	110.0	**185.8**	102.6
Itpm2	**33.75**	18.89	**39.78**	9.737	**77.87**	41.27	**48.44**	47.86
Ipcm1	63.33	**76.67**	**105.7**	44.79	**74.63**	42.33	**86.26**	68.95
Ipcm6	85.42	**111.9**	**147.1**	81.79	**173.3**	125.4	**203.7**	170.9
Ivlm1	86.25	-	**88.83**	48.69	**98.64**	57.14	**72.33**	69.52
Ivlm3	**146.7**	94.44	**154.2**	126.6	**176.5**	101.1	**125.4**	102.0
Ivlm7	**109.2**	92.59	**141.7**	100.3	**173.3**	172.0	-	-
Iscm1	**82.50**	71.85	**149.9**	81.79	**126.5**	96.30	111.5	**124.2**
Iscm4	47.92	**53.70**	87.19	**89.09**	**79.17**	43.39	**96.22**	80.29
	49.17		64.31					50.72
Iscm6	-	-	-	-	**102.5**	-	**74.32**	51.85
Mesothorax								
IIdlm1	**492.5**	405.6	**736.8**	481.0	**908.5**	639.2	**969.5**	717.9
IIdlm2	**291.7**	244.8	**427.8**	311.1	**550.3**	420.6	**613.8**	423.4
							**583.9**	
IIdlm3	-	-	**93.19**	39.92	**129.8**	94.18	**136.0**	62.68
IIdvm2	**425.4**	321.5	**598.4**	395.3	**686.6**	507.9	**775.0**	566.4
IIdvm4	**404.6**	330.4	**583.1**	434.3	**675.5**	551.3	**756.5**	531.1
IIdvm7	**563.8**	441.1	**612.0**	446.9	**686.6**	538.6	**784.3**	622.2
IItpm1	**85.42**	55.92	**112.3**	73.03	**144.1**	98.94	**147.3**	107.7
IItpm2	52.92	-	**115.0**	55.99	**103.8**	101.6	**109.5**	85.47
IItpm3	**120.0**	68.89	**112.8**	92.99	148.0	**174.1**	**319.2**	144.2
IItpm4	**86.25**	71.85	**146.0**	86.17	**135.0**	117.5	**172.5**	132.2
IItpm5	**108.8**	94.81	**157.5**	102.2	**136.3**	116.4	**185.1**	132.2
					82.41			110.5
IItpm6	**100.8**	90.74	**173.3**	107.1	**175.9**	121.7	**185.8**	125.9
IItpm7	**112.5**	107.4	**173.3**	117.8	**253.1**	165.1	**217.7**	143.6
IItpm8	**96.25**	70.74	88.28	**92.99**	**172.0**	105.8	**199.1**	148.7
			**106.3**	74.00		132.3		
IItpm9	63.33	**89.26**	**117.3**	88.61	**203.1**	150.8	**173.9**	115.1
			**122.2**	103.2				
IIspm2	**112.5**	74.07	**136.2**	124.1	**162.2**	105.8	**152.6**	108.3
IIpcm6	**142.1**	107.8	**233.2**	160.7	**305.6**	206.9	**331.8**	223.4
IIvlm3	15.83	-	**115.0**	42.84	**183.0**	91.01	**144.7**	120.8
IIscm1	**115.0**	78.15	**137.9**	100.8	**196.0**	141.8	**180.5**	115.7
IIscm2	**146.3**	109.3	**180.4**	126.6	**199.9**	131.2	**199.7**	177.8
IIscm6	**153.3**	101.1	**211.4**	74.00	**170.0**	32.28	160.6	**161.8**
	**132.5**		**178.2**	117.8	**142.8**	96.30	151.3	**181.7**
tendon	67.50	**70.74**	**213.6**	144.1	-	-	-	-
Metathorax								
IIIspm1	96.25	**110.4**	**159.1**	128.0	169.4	**222.2**	**242.2**	172.1
IIIspm2	-	-	82.83	-	72.03	**101.6**	**127.4**	109.4
IIIspm5	-	-	125.3	-	159.0	**168.3**	**167.9**	90.03
IIIpcm3	-	202.6	-	200.1	**154.4**	129.6	**193.8**	143.6
IIIpcm6	**190.8**	88.15	**218.0**	166.0	**234.3**	218.0	169.2	**257.0**
IIIvlm2	-	-	168.9	-	149.3	-	**170.5**	92.88
IIIscm1	-	-	**152.6**	95.42	**115.5**	93.65	**119.4**	67.24
IIIscm2	66.67	**124.8**	**230.0**	82.28	**176.5**	158.7	**209.0**	137.9
IIIscm6	**205.0**	182.2	**389.6**	297.0	**266.7**	185.7	**260.1**	206.8

**Table 6:** The absolute widths (µm) of thoracic muscles in each period. Within the same sex, the absolute width broader than that in the previous developmental period is denoted in blue, while the narrower width is denoted in yellow. In the same period, the broader relative width is denoted in bold. Absent is denoted with “-”.

	PD3		PD4		AD1		AD3	
Muscle	Female	Male	Female	Male	Female	Male	Female	Male
Prothorax								
Idlm1	8.333	-	16.35	-	**29.20**	12.17	17.92	**22.79**
Idlm2	**32.92**	32.08	**92.10**	44.30	**118.1**	14.81	**19.91**	17.09
Idlm4	-	-	**-**	-	-	19.58	23.22	**25.07**
Idvm2	13.75	-	**73.57**	14.61	**64.89**	15.34	**66.36**	39.89
Idvm3	13.33	**24.17**	**35.42**	19.96	24.01	**52.91**	59.72	**97.44**
Idvm19	17.08	**20.83**	**38.15**	18.99	**38.94**	22.22	**54.41**	38.18
Itpm1	**40.42**	19.58	**64.31**	22.40	**50.62**	21.16	**31.19**	30.20
Itpm2	28.33	**40.42**	**101.9**	45.28	**125.2**	83.60	**70.34**	63.25
Ipcm1	24.17	**33.75**	**45.23**	25.8	**47.37**	37.04	**43.13**	33.62
Ipcm6	25.42	**29.58**	**27.25**	13.63	**39.58**	37.04	31.85	**41.03**
Ivlm1	10.42	-	**23.43**	23.37	**19.47**	12.17	**19.24**	13.68
Ivlm3	24.58	**40.00**	**62.67**	54.04	**55.81**	44.97	27.21	**27.35**
Ivlm7	29.58	**39.17**	**75.20**	38.46	46.72	**57.14**	-	-
Iscm1	**45.83**	35.42	**85.01**	48.69	**111.6**	47.09	78.30	**101.4**
Iscm4	**28.75**	14.58	**52.32**	24.83	**32.45**	15.34	39.81	39.81
	**38.33**		**52.86**					**53.75**
Iscm6	-	-	-	-	64.89	-	**32.51**	29.06
Mesothorax								
IIdlm1	**228.3**	208.8	**261.0**	195.7	**395.8**	263.5	**416.7**	284.9
IIdlm2	**100.3**	95.83	**179.8**	132.4	**202.5**	119.0	99.54	**148.1**
							146.6	
IIdlm3	**-**	-	**48.50**	31.65	**53.21**	48.68	**57.73**	30.77
IIdvm2	**100.0**	85.42	**134.6**	83.25	**149.3**	118.0	**176.5**	101.4
IIdvm4	**67.5**	45.83	92.64	**93.48**	**104.5**	76.72	**114.1**	85.47
IIdvm7	**100.0**	71.25	**191.3**	121.2	**206.4**	137.6	**238.9**	153.8
IItpm1	36.25	**37.50**	**65.40**	38.95	**61.65**	38.10	**106.2**	55.27
IItpm2	79.17	-	**103.5**	64.26	**135**	75.13	**128.7**	96.87
IItpm3	**25.83**	17.08	**29.43**	19.47	**27.26**	21.16	**33.18**	27.92
IItpm4	47.08	**54.58**	**94.82**	48.69	**97.34**	59.26	**142.7**	91.74
IItpm5	29.17	29.17	**48.50**	24.34	45.43	**47.09**	**74.32**	45.58
					46.07			39.89
IItpm6	12.50	**25.83**	**54.50**	20.45	**46.07**	37.04	**53.09**	51.28
IItpm7	**27.08**	28.33	**58.31**	38.46	**73.98**	40.74	**124.1**	63.82
IItpm8	**20.83**	18.75	**53.95**	24.34	**51.91**	48.69	**43.13**	14.25
			**21.80**	12.17		13.63		
IItpm9	21.67	**52.92**	**42.84**	19.96	**59.05**	45.50	80.96	**106.6**
			**48.69**	25.32				
IIspm2	83.33	**88.33**	**180.9**	97.37	**162.2**	137.0	**199.1**	135.6
IIpcm6	46.25	**67.50**	**99.18**	82.77	**95.39**	91.01	102.9	**106.6**
IIvlm3	13.33	-	**49.05**	20.45	17.52	**27.51**	18.58	**19.94**
IIscm1	**27.08**	26.25	**49.59**	30.67	**66.19**	42.33	**87.59**	46.72
IIscm2	24.17	**27.5**	**101.4**	28.24	**59.70**	11.64	**54.41**	45.58
IIscm6	**25.83**	16.25	72.48	**88.12**	72.68	**137.0**	82.28	**104.3**
	15.42		**41.42**	19.47	29.85	**91.01**	28.53	**63.25**
tendon	**18.75**	17.50	**61.04**	18.50	-	-	-	-
Metathorax								
IIIspm1	12.92	**17.92**	23.43	18.50	**18.82**	13.23	**19.24**	12.54
IIIspm2	-	-	61.04	-	**59.70**	11.11	13.93	**51.85**
IIIspm5	-	-	44.14	-	**77.87**	62.96	**59.72**	40.46
IIIpcm3	-	15.83	-	25.32	**25.96**	21.16	19.24	**23.36**
IIIpcm6	12.50	**19.17**	**27.25**	22.88	**31.15**	30.69	48.44	**63.82**
IIIvlm2	-	-	27.25	-	**51.91**	-	**63.04**	34.19
IIIscm1	-	-	**59.95**	33.11	**92.80**	62.43	34.51	**102.6**
IIIscm2	**45.42**	27.92	**65.94**	15.09	**124.6**	39.68	**78.30**	63.25
IIIscm6	28.33	**28.75**	**48.50**	21.91	**61.65**	27.51	**60.38**	47.29

**Table 7:** The relative lengths of thoracic muscles in each period. Within the same sex, the relative length longer than that in the previous developmental period is denoted in blue, while the shorter length is denoted in yellow. In the same period, the longer relative length is denoted in bold. Absent is denoted with “-”.

	PD3		PD4		AD1		AD3	
Muscle	Female	Male	Female	Male	Female	Male	Female	Male
Prothorax								
Idlm1	131.1	-	201.3	-	**177.0**	120.0	144.3	**150.4**
Idlm2	**309.5**	259.3	**320.0**	296.3	**345.6**	274.1	**262.3**	170.4
Idlm4	-	-	-	-	-	120.0	**98.36**	81.48
Idvm2	146.2	-	**146.9**	106.7	**105.6**	74.07	**157.4**	157.0
Idvm3	80.00	**116.3**	**152.1**	93.33	55.74	**172.6**	119.3	**201.5**
Idvm19	**480.0**	394.8	**376.4**	318.5	323.3	**326.7**	329.8	**370.4**
Itpm1	**182.3**	177.8	**137.7**	127.4	**158.7**	154.1	**183.6**	133.3
Itpm2	**53.11**	37.78	**47.87**	14.81	**78.69**	57.78	47.87	**62.22**
Ipcm1	99.67	**153.3**	**127.2**	68.15	**75.41**	59.26	85.25	**89.63**
Ipcm6	134.4	**223.7**	**177.0**	124.4	175.1	**175.6**	201.3	**222.2**
Ivlm1	135.7	-	**106.9**	74.07	**99.67**	80.00	71.48	**90.37**
Ivlm3	**230.8**	188.9	185.6	**192.6**	**178.4**	141.5	123.9	**132.6**
Ivlm7	171.8	**185.2**	**170.5**	152.6	175.1	**240.7**	-	-
Iscm1	129.8	**143.7**	**180.3**	124.4	127.9	**134.8**	110.2	**161.5**
Iscm4	75.41	**107.4**	104.9	**135.6**	**80.00**	60.74	**95.08**	79.34
	77.38		77.37					59.02
Iscm6	-	-	-	-	103.6	-	**73.44**	67.41
Mesothorax								
IIdlm1	775.1	**811.1**	**886.6**	731.9	**918.0**	894.8	**958.0**	933.3
IIdlm2	459.0	**489.6**	**514.8**	473.3	556.1	**588.9**	**606.6**	550.4
							**577.0**	
IIdlm3	-	-	**112.1**	60.74	131.1	**131.9**	**134.4**	81.48
IIdvm2	**669.5**	643.0	**720.0**	601.5	693.8	**711.1**	**765.9**	736.3
IIdvm4	636.7	**660.7**	**701.6**	660.7	682.6	**771.9**	**747.5**	690.4
IIdvm7	**887.2**	882.2	**736.4**	680.0	693.8	**754.1**	775.1	**808.9**
IItpm1	**134.4**	111.9	**135.1**	111.1	**145.6**	138.5	**145.6**	140.0
IItpm2	83.28	-	**138.4**	85.19	104.9	**142.2**	108.2	**111.1**
IItpm3	**188.9**	137.8	135.7	**141.5**	149.5	**243.7**	**315.4**	187.4
IItpm4	135.7	**143.7**	**175.7**	131.1	136.4	**164.4**	170.5	**171.9**
IItpm5	171.1	**189.6**	**189.5**	155.6	137.7	**163.0**	**183.0**	171.9
					83.28			143.7
IItpm6	158.7	**181.5**	**208.5**	163.0	**177.7**	170.4	**183.6**	163.7
IItpm7	177.0	**214.8**	**208.5**	179.3	**255.7**	231.1	**215.1**	186.7
IItpm8	**151.5**	141.5	106.2	**141.5**	173.8	148.1	**196.7**	193.3
			**127.9**	112.6		**185.2**		
IItpm9	99.67	**178.5**	**178.5**	134.8	205.2	**211.1**	**171.8**	149.6
			**185.9**	157.0				
IIspm2	**177.0**	148.1	163.9	**188.9**	**163.9**	148.1	**150.8**	140.7
IIpcm6	**223.6**	215.6	**280.7**	244.4	**308.9**	289.6	**327.9**	290.4
IIvlm3	24.92	-	**138.4**	65.19	**184.9**	127.4	143.0	**157.0**
IIscm1	**181.0**	156.3	**165.9**	153.3	198.0	**198.5**	**178.4**	150.4
IIscm2	**230.2**	218.5	**217.0**	192.6	**202.0**	183.7	197.4	**231.1**
IIscm6	**241.3**	202.2	**254.4**	112.6	**171.8**	45.19	158.7	**210.4**
	**208.5**		**214.4**	179.3	**144.3**	134.8	149.5	**236.3**
tendon	106.2	**141.5**	**257.1**	219.3	-	-	-	-
Metathorax								
IIIspm1	151.5	**220.7**	191.5	**194.8**	171.1	**311.1**	**239.3**	223.7
IIIspm2	-	-	99.67	-	72.79	**142.2**	125.9	**142.2**
IIIspm5	-	-	150.8	-	160.7	**235.6**	**165.9**	117.0
IIIpcm3	-	405.2	-	304.4	156.1	**181.5**	**191.5**	186.7
IIIpcm6	**300.3**	176.3	**262.3**	252.6	236.7	**305.2**	167.2	**334.1**
IIIvlm2	-	-	203.3	-	150.8	-	**168.5**	120.7
IIIscm1	-	-	**183.6**	145.2	116.7	**131.1**	**118.0**	87.41
IIIscm2	104.9	**249.6**	**276.7**	125.2	178.4	**222.2**	**206.6**	179.3
IIIscm6	322.6	**364.4**	**468.9**	451.9	**269.5**	260.0	257.0	**268.9**

**Table 8:** The relative widths of thoracic muscles in each period. Within the same sex, the relative width broader than that in the previous developmental period is denoted in blue, while the narrower width is denoted in yellow. In the same period, the broader relative width is denoted in bold. Absent is denoted with “-”.

	PD3		PD4		AD1		AD3	
Muscle	Female	Male	Female	Male	Female	Male	Female	Male
Prothorax								
Idlm1	13.11	-	19.67	-	**29.51**	17.04	17.70	**29.63**
Idlm2	51.80	**57.04**	**110.8**	67.41	**119.3**	20.74	19.67	**22.22**
Idlm4	-	-	-	-	-	27.41	22.95	**32.59**
Idvm2	21.64	-	**88.52**	22.22	**65.57**	21.48	**65.57**	51.85
Idvm3	20.98	**42.96**	**42.62**	30.37	24.26	**74.07**	59.02	**126.7**
Idvm19	26.89	**37.04**	**45.90**	28.89	**39.34**	31.11	**53.77**	49.63
Itpm1	**63.61**	34.81	**77.38**	34.07	**51.15**	29.63	30.82	**39.26**
Itpm2	44.59	**71.85**	**122.6**	68.89	**126.6**	117.0	69.51	**82.22**
Ipcm1	38.03	**60.00**	**54.43**	39.26	47.87	**51.85**	42.62	**43.70**
Ipcm6	40.00	**52.59**	**32.79**	20.74	40.00	**51.85**	31.48	**53.33**
Ivlm1	16.39	-	28.20	**35.56**	**19.67**	17.04	**19.02**	17.78
Ivlm3	38.69	**71.11**	75.41	**82.22**	56.39	**62.96**	26.89	**35.56**
Ivlm7	46.56	**69.63**	**90.49**	58.52	47.21	**80.00**	-	-
Iscm1	**72.13**	62.96	**102.3**	74.07	**112.8**	65.93	77.38	**131.9**
Iscm4	**45.25**	25.93	**62.95**	37.78	**32.79**	21.48	39.34	39.34
	**60.33**		**63.61**					**53.11**
Iscm6	-	-	-	-	65.57	-	32.13	**37.78**
Mesothorax								
IIdlm1	359.3	**371.1**	**314.1**	297.8	**400.0**	368.9	**411.8**	370.4
IIdlm2	158.7	**170.4**	**216.4**	201.5	**204.6**	166.7	98.36	**192.6**
							57.05	
IIdlm3	-	-	**58.36**	48.15	53.77	**68.15**	**174.4**	40.00
IIdvm2	**157.4**	151.9	**162.0**	126.7	150.8	**165.2**	112.8	**131.9**
IIdvm4	57.05	**81.48**	111.5	**142.2**	105.6	**107.4**	**236.1**	111.1
IIdvm7	124.6	**126.7**	**230.2**	184.4	**208.5**	192.6	104.9	**200.0**
IItpm1	40.66	**66.67**	**78.69**	59.26	**62.30**	53.33	**127.2**	71.85
IItpm2	74.10	-	**124.6**	97.78	**136.4**	105.2	32.79	**125.9**
IItpm3	**45.90**	30.37	**35.41**	29.63	27.54	**29.63**	**141.0**	36.30
IItpm4	19.67	**97.04**	**114.1**	74.07	**98.36**	82.96	**174.4**	119.3
IItpm5	42.62	**51.85**	**58.36**	37.04	45.90	**65.93**	**73.44**	59.26
					46.56			51.85
IItpm6	32.79	**45.93**	**65.57**	31.11	46.56	**51.85**	52.46	**66.67**
IItpm7	34.10	**50.37**	**70.16**	58.52	**74.75**	57.04	**122.6**	82.96
IItpm8	131.1	**33.33**	**64.92**	37.04	52.46	**74.07**	**42.62**	18.52
			**26.23**	18.52		20.74		
IItpm9	72.79	**94.07**	**65.19**	30.37	59.67	**63.70**	80.00	**138.5**
			**74.07**	38.52				
IIspm2	131.1	**157.0**	**217.7**	148.1	163.9	**191.9**	**196.7**	176.3
IIpcm6	72.79	**120.0**	119.3	**125.9**	96.39	**127.4**	101.6	**138.5**
IIvlm3	20.98	-	**59.02**	31.11	17.70	**38.52**	18.36	**25.93**
IIscm1	42.62	**46.67**	**59.67**	46.67	**66.89**	59.26	**86.56**	60.74
IIscm2	38.03	**48.89**	**122.0**	42.96	**60.33**	16.30	53.77	**59.26**
IIscm6	**40.66**	28.89	87.21	**134.1**	**73.44**	28.15	81.31	**135.6**
	24.26		**49.84**	29.63	30.16	**65.93**	28.20	**82.22**
tendon	29.51	**31.11**	**73.44**	28.15	-	-	-	-
Metathorax								
IIIspm1	20.33	**31.85**	**28.20**	28.15	**19.02**	18.52	**19.02**	16.30
IIIspm2	-	-	73.44	-	**60.33**	15.56	13.77	**67.41**
IIIspm5	-	-	53.11	-	78.69	**88.15**	**59.02**	52.59
IIIpcm3	-	28.15	-	38.52	26.23	**29.63**	19.02	**30.37**
IIIpcm6	19.67	**34.07**	32.79	**34.81**	31.48	**42.96**	47.87	**82.96**
IIIvlm2	-	-	32.79	-	52.46	-	**62.30**	44.44
IIIscm1	-	-	72.13	50.37	**93.77**	87.41	34.10	**133.3**
IIIscm2	**71.48**	49.63	**79.34**	22.96	**125.9**	55.56	77.38	**82.22**
IIIscm6	44.59	**51.11**	**58.36**	33.33	**62.30**	38.52	59.67	**61.48**

## Data Availability

The data presented in this study are available upon reasonable request from the corresponding author.

## Supplementary Materials

The following supporting information can be downloaded at: https://www.mdpi.com/article/10.3390/insects14110893/s1, Video S1: PD3 Female; Video S2: PD3 Male; Video S3: PD4 Female; Video S4: PD4 Male; Video S5: AD1 Female; Video S6: AD1 Male; Video S7: AD3 Female; Video S8: AD3 Male.

## References

[1] Engel M.S. (2015). Insect evolution. Curr. Biol..

[2] Hall M.J.R., Martín-Vega D. (2019). Visualization of insect metamorphosis. Philos. Trans. R. Soc. B.

[3] Rolff J., Johnston P.R., Reynolds S. (2019). Complete metamorphosis of insects. Philos. Trans. R. Soc. B.

[4] Jindra M., Palli S.R., Riddiford L.M. (2012). The juvenile hormone signaling pathway in insect development. Annu. Rev. Entomol..

[5] Lee G., Park J.H. (2021). Programmed cell death reshapes the central nervous system during metamorphosis in insects. Curr. Opin. Insect Sci..

[6] Zhao C.J., Ang Y.C., Wang M.Q., Gao C.X., Zhang K.Y., Tang C.F., Liu X., Li M., Yang D., Meier R. (2020). Contribution to understanding the evolution of holometaboly: Transformation of internal head structures during the metamorphosis in the green lacewing *Chrysopa pallens* (Neuroptera: Chrysopidae). BMC Evol. Biol..

[7] Nel A., Roques P., Nel P., Prokop J., Steyer J.S. (2007). The earliest holometabolous insect from the Carboniferous: A “crucial” innovation with delayed success (Insecta Protomeropina Protomeropidae). Ann. Société Entologique Fr..

[8] Truman J.W., Riddiford L.M. (2019). The evolution of insect metamorphosis: A developmental and endocrine view. Philos. Trans. R. Soc. B.

[9] Stephenson R., Metcalfe N.H. (2013). *Drosophila melanogaster*: A fly through its history and current use. J. R. Coll. Physicians Edinb..

[10] Gillette C.M., Tennessen J.M., Reis T. (2021). Balancing energy expenditure and storage with growth and biosynthesis during *Drosophila* development. Dev. Biol..

[11] De Celis J.F. (2003). Pattern formation in the *Drosophila* wing: The development of the veins. Bioessays.

[12] De la Loza M.C., Thompson B.J. (2017). Forces shaping the *Drosophila* wing. Mech. Dev..

[13] Liu S.N., Sun J., Wang D., Pflugfelder G.O., Shen J. (2016). Fold formation at the compartment boundary of *Drosophila* wing requires Yki signaling to suppress JNK dependent apoptosis. Sci. Rep..

[14] Robertson C.W. (1936). The metamorphosis of *Drosophila melanogaster*, including an accurately timed account of the principal morphological changes. J. Morphol..

[15] Bainbridge S.P., Bownes M. (1981). Staging the metamorphosis of *Drosophila melanogaster*. J. Embryol. Exp. Morphol..

[16] Bodenstein D., Demerec M. (1950). The postembryonic development of *Drosophila*. Biology of Drosophila.

[17] Hartenstein V., Bate M., Arias A.M. (1993). Atlas of *Drosophila* development. The Development of Drosophila Melanogaster.

[18] Zalokar M. (1947). Anatomie du thorax de *Drosophila melanogaster*. Rev. Suisse Zool..

[19] Ferris G.F., Demerec M. (1950). External morphology of the adult. Biology of Drosophila.

[20] Miller A., Demerec M. (1950). The internal anatomy and histology of the imago of *Drosophila melanogaster*. Biology of Drosophila.

[21] Fabian B., Schneeberg K., Beutel R.G. (2016). Comparative thoracic anatomy of the wild type and *wingless* (wg^1^cn^1^) mutant of *Drosophila melanogaster* (Diptera). Arthropod Struct. Dev..

[22] Wiplfer B., Schneeberg K., Löffler A., Hünefeld F., Meier R., Beutel R.G. (2013). The skeletomuscular system of the larva of *Drosophila melanogaster* (Drosophilidae, Diptera)—A contribution to the morphology of a model organism. Arthropod Struct. Dev..

[23] Gramates L.S., Agapite J., Attrill H., Calvi B.R., Crosby M., dos Stantos G., Goodman J.L., Goutte-Gattat D., Jenkins V., Kaufman T. (2022). FlyBase Concortium. FlyBase: A guided tour of highlighted features. Genetics.

[24] Friedrich F., Beutel R.G. (2008). The thorax of *Zorotypus* (Hexapoda, Zoraptera) and a new nomenclature for the musculature of Neoptera. Arthropod Struct. Dev..

[25] Court R., Namiki S., Armstrong D., Börner J., Card G., Costa M., Dickinson M., Duch C., Korff W., Mann R. (2020). A systematic nomenclature for the *Drosophila* ventral nerve cord. Neuron.

[26] Friedrich F., Matsumura Y., Pohl H., Bai M., Hörnschemeyer T., Beutel R.G. (2014). Insect morphology in the age of phylogenomics: Innovative techniques and its future role in systematics. Entomol. Sci..

[27] Zhao C.J., Wang M.Q., Gao C.X., Li M., Zhang K.Y., Yang D., Liu X. (2022). Evolution of holometaboly revealed by developmental transformation of internal thoracic structures in a green lacewing *Chrysopa pallens* (Neuroptera: Chrysopidae). Insect Sci..

[28] Fernandes J.J., Keshishian H. (1996). Patterning the dorsal longitudinal flight muscles (DLM) of *Drosophila*: Insights from the ablation of larval scaffolds. Development.

[29] Ludwig J.C., Trimmer B.A. (2021). Metamorphosis in insect muscle: Insight for engineering muscle-based actuator. Tissue Eng. Part B Rev..

[30] Beutel R.G., Friedrich F., Ge S.Q., Yang X.K. (2014). 1.2.3 Cephalic endoskeleton. Insect Morphology and Phylogeny: A Text Book for Students of Entomology.

[31] Ready N.E., Josephson R.K. (1982). Flight muscle development in a hemimetabolous insect. J. Exp. Zool..

[32] Martín-Vega D., Simonsen T.J., Hall M.J.R. (2017). Looking into the puparium: Micro-CT visualization of the internal morphological changes during metamorphosis of the blow fly, *Calliphora vicina*, with the first quantitative analysis of organ development in cyclorrhaphous dipterans. J. Morphol..

[33] Helm B.R., Payne S., Rinehart J.P., Yocum G.D., Bowsher J.H., Greenlee K.J. (2018). Micro-computed tomography of pupal metamorphosis in the solitary bee *Megachile rotundata*. Arthropod Struct. Dev..

[34] Ellington C.P. (1985). Power and efficiency of insect flight muscle. J. Exp. Biol..

[35] Deora T., Gundish N., Sane S.P. (2017). Mechanics of the thorax in flies. J. Exp. Biol..

[36] Lihoreau M., Poissonnier L.-A., Isabel G., Dussutour A. (2016). *Drosophila* female trade off good nutrition with high-quality oviposition sites when choosing foods. J. Exp. Biol..

[37] Schulman V.K., Dobi K.C., Baylies M.K. (2016). Morphogenesis of the somatic musculature in *Drosophila melanogaster*. Wiley Interdiscip. Rev. Dev. Biol..

[38] Brodsky A.K. (1994). The Evolution of Insect Flight.

[39] Heming B.S. (2003). Insect Development and Evolution.

[40] Reaume C.J., Sokolowski M.B. (2006). The nature of *Drosophila melanogaster*. Curr. Biol..

[41] Truman J.W., Schuppe H., Shepherd D., Williams D.W. (2004). Developmental architecture of adult-specific lineages in the ventral CNS of Drosophila. Development.

[42] Azevedo A.W., Lesser E., Mark B., Phelps J.S., Elabbady L., Kuroda S., Sustar A.E., Moussa A.J., Kandelwal A., Dallmann C.J. (2022). Tools for comprehensive reconstruction and analysis of *Drosophila* motor circuits. bioRxiv.

[43] Takemura S.Y., Hayworth K.J., Huang G.B., Januszewski M., Lu Z., Marin E.C., Preibisch S., Xu C.S., Bogovic J., Champion A.S. (2023). A connectome of the male *Drosophila* ventral nerve cord. bioRxiv.

